# Synergistic Anti-arrhythmic Effects in Human Atria with Combined Use of Sodium Blockers and Acacetin

**DOI:** 10.3389/fphys.2017.00946

**Published:** 2017-11-23

**Authors:** Haibo Ni, Dominic G. Whittaker, Wei Wang, Wayne R. Giles, Sanjiv M. Narayan, Henggui Zhang

**Affiliations:** ^1^Biological Physics Group, University of Manchester, Manchester, United Kingdom; ^2^Space Institute of Southern China, Shenzhen, China; ^3^Key Laboratory of Medical Electrophysiology, Ministry of Education, Collaborative Innovation Center for Prevention and Treatment of Cardiovascular Disease/Institute of Cardiovascular Research, Southwest Medical University, Luzhou, China; ^4^Faculties of Kinesiology and Medicine, University of Calgary, Calgary, AB, Canada; ^5^Department of Medicine, Stanford University School of Medicine, Stanford, CA, United States; ^6^School of Computer Science and Technology, Harbin Institute of Technology, Harbin, China

**Keywords:** atrial-selective block, atrial fibrillation, sodium and potassium current block, multiscale simulation, synergistic antiarrhythmic effect

## Abstract

Atrial fibrillation (AF) is the most common cardiac arrhythmia. Developing effective and safe anti-AF drugs remains an unmet challenge. Simultaneous block of both atrial-specific ultra-rapid delayed rectifier potassium (K^+^) current (I_Kur_) and the Na^+^ current (I_Na_) has been hypothesized to be anti-AF, without inducing significant QT prolongation and ventricular side effects. However, the antiarrhythmic advantage of simultaneously blocking these two channels vs. individual block in the setting of AF-induced electrical remodeling remains to be documented. Furthermore, many I_Kur_ blockers such as acacetin and AVE0118, partially inhibit other K^+^ currents in the atria. Whether this multi-K^+^-block produces greater anti-AF effects compared with selective I_Kur_-block has not been fully understood. The aim of this study was to use computer models to (i) assess the impact of multi-K^+^-block as exhibited by many I_Kur_ blokers, and (ii) evaluate the antiarrhythmic effect of blocking I_Kur_ and I_Na_, either alone or in combination, on atrial and ventricular electrical excitation and recovery in the setting of AF-induced electrical-remodeling. Contemporary mathematical models of human atrial and ventricular cells were modified to incorporate dose-dependent actions of acacetin (a multichannel blocker primarily inhibiting I_Kur_ while less potently blocking I_to_, I_Kr_, and I_Ks_). Rate- and atrial-selective inhibition of I_Na_ was also incorporated into the models. These single myocyte models were then incorporated into multicellular two-dimensional (2D) and three-dimensional (3D) anatomical models of the human atria. As expected, application of I_Kur_ blocker produced pronounced action potential duration (APD) prolongation in atrial myocytes. Furthermore, combined multiple K^+^-channel block that mimicked the effects of acacetin exhibited synergistic APD prolongations. Synergistically anti-AF effects following inhibition of I_Na_ and combined I_Kur_/K^+^-channels were also observed. The attainable maximal AF-selectivity of I_Na_ inhibition was greatly augmented by blocking I_Kur_ or multiple K^+^-currents in the atrial myocytes. This enhanced anti-arrhythmic effects of combined block of Na^+^- and K^+^-channels were also seen in 2D and 3D simulations; specially, there was an enhanced efficacy in terminating re-entrant excitation waves, exerting improved antiarrhythmic effects in the human atria as compared to a single-channel block. However, in the human ventricular myocytes and tissue, cellular repolarization and computed QT intervals were modestly affected in the presence of actions of acacetin and I_Na_ blockers (either alone or in combination). In conclusion, this study demonstrates synergistic antiarrhythmic benefits of combined block of I_Kur_ and I_Na_, as well as those of I_Na_ and combined multi K^+^-current block of acacetin, without significant alterations of ventricular repolarization and QT intervals. This approach may be a valuable strategy for the treatment of AF.

## Introduction

Despite recent advances in the management of Atrial fibrillation (AF), the world's most common cardiac arrhythmia (Dobrev et al., 2012; Nattel and Dobrev, 2017), developing effective and safe antiarrhythmic drugs for treatment of AF remains challenging (Aguilar-Shardonofsky et al., 2012; Aguilar et al., 2015). Frequently these antiarrhythmic agents promote ventricular arrhythmias (Dobrev et al., 2012; Woods and Olgin, 2014; Voigt and Dobrev, 2016) by prolonging cellular action potential durations (APDs). The associated QT-interval prolongation can lead to life-threatening consequences. Developing atrial-selective drugs is acknowledged to be a current strategy for the treatment of AF (Burashnikov et al., 2007).

Atrial and ventricular tissues show intrinsic regional differences in their cellular ion channel properties, thus suggesting a basis for developing atrial-selective drugs. For example, the atrial and ventricular fast sodium (Na^+^) channel currents (I_Na_) exhibit different voltage-dependent inactivation properties, opening the opportunity for atrial-selective Na^+^ channel blockade (Burashnikov et al., 2007; Antzelevitch and Burashnikov, 2009; Zygmunt et al., 2011). Previous simulation studies have demonstrated that by optimizing state-dependent Na^+^-channel blocking dynamics (i.e., drug-channel interaction parameters), atrial-selective block of I_Na_ could be achieved and that could maximize pharmaceutical effects on the atria while minimizing their proarrhythmic actions in the ventricles (Aguilar-Shardonofsky et al., 2012; Aguilar et al., 2015).

Another tissue-specific difference between the atria and ventricles is that the ultra-rapid delayed rectifier potassium current (I_Kur_, carried by the K_V_1.5 channel) contributes to repolarization in the atria but plays little role in the ventricles (Tamargo et al., 2009; Ravens and Wettwer, 2011). Recent studies suggest that atrial-selective blockade of I_Kur_ may be an effective pharmacological treatment of AF (Li et al., 2008; Pavri et al., 2012; Loose et al., 2014; Ford et al., 2016). Although the efficacy of I_Kur_ block in the treatment of AF remains controversial (Burashnikov and Antzelevitch, 2008), multiple I_Kur_ blockers have been developed (Tamargo et al., 2009; Loose et al., 2014; Wettwer and Terlau, 2014; Ford et al., 2016). Interestingly, these I_Kur_ blockers actually target multiple channels, and are known to inhibit other K^+^ currents including I_to_ and I_K, ACh_ in the atria (Burashnikov and Antzelevitch, 2008). Examples of such blockers include AVE0118 (Gögelein et al., 2004), AVE1231 (Wirth et al., 2007), AZD7009 (Persson et al., 2005), and acacetin (Li et al., 2008). Among these channel blockers, acacetin, a natural flavone initially isolated from a traditional Chinese medicine *Xuelianhua*, potently blocks I_Kur_, I_to_, and I_K,ACh_, and has a smaller potency in inhibiting I_Kr_ and I_Ks_ (Li et al., 2008), similar to AVE0118 (Gögelein et al., 2004; Haan et al., 2006). Acacetin is regarded as a promising atrial-selective agent for the treatment of AF (Li et al., 2008). However, the actions of acacetin on atrial electrophysiology, especially its effects following AF-induced electrical remodeling of atrial electrophysiological properties (Dobrev et al., 2012), remain to be elucidated. Furthermore, since most I_Kur_ blockers inhibit other K^+^ channels, the question whether the “additional” inhibitive actions produce favorable antiarrhythmic effects has not been addressed thoroughly. A better understanding of these effects of modulating multiple ion channels on atrial excitation and recovery/repolarization may provide insights into evaluating and developing antiarrhythmic drugs.

Interestingly, simultaneous multiple-channel blocking of both depolarization and repolarization currents is attracting more attention since empirical observations suggest that such multi-channel blockers generally mediate more effective antiarrhythmic effects (Kirchhoff et al., 2015; Reiffel et al., 2015; Hartmann et al., 2016). A recent numerical and experimental study on the canine heart (Aguilar et al., 2015) suggested that blocking K^+^ currents enhanced the anti-arrhythmic effects and AF-selectivity of I_Na_ blockade. In their study, I_Kur_ block was modeled using a simple pore block scheme by reducing the conductance of the channel. As the kinetics of drug action plays an important role in the effects of I_Kur_ blockers (Scholz et al., 2013; Ellinwood et al., 2017), in simulating I_Kur_ block a state-dependent block model reproducing a realistic blocker is more favorable. Once again, the effects of combined I_Na_ and I_Kur_ block on the human atria, especially in the setting of AF-induced electrical remodeling which reduced I_Kur_, remain to be elucidated. It is also unclear how multiple-channel blockade may affect QT interval.

In the present study, it was hypothesized that combined block of I_Na_ and K^+^-currents (predominantly I_Kur_) could produce antiarrhythmic benefits compared with the application of either blocker alone in the setting of AF-induced electrical remodeling. We have tested the hypothesis with the following three aims: (i) to identify and illustrate the effects of the realistic I_Kur_ blocker, acacetin, on atrial electrophysiology following AF-related remodeling; (ii) to assess whether combined I_Na_ and I_Kur_ block produce synergistic antiarrhythmic effects; and (iii) to investigate the action of such drug combinations on ventricular electrophysiology.

## Methods

### Modeling electrophysiology of the human heart

To simulate human atrial electrophysiology, an updated Colman et al. model for atrial electrophysiology (Colman et al., 2013, 2017) was used. For *in silico* study of effects of chronic AF- (cAF) induced electrophysiological remodeling on the atria, we incorporated the cAF model parameters from our previous study (Colman et al., 2013) into the updated atrial single cell model (for details please see Online Supplement Material 1.1).

To assess the effects of the anti-AF drugs on the human ventricles, simulations were performed to investigate the actions of the anti-AF drugs on the ventricular AP, I_Na_ and QT intervals in the electrograms. In these simulations, the mathematical model developed by O'Hara et al. (2011) was used to represent the ventricular electrophysiology. Additionally, the I_Na_ formulation in the model was replaced by the one in the Luo–Rudy model (Luo and Rudy, 1994), which enabled electrical excitation to propagate in the tissue model.

More detailed descriptions of the electrophysiological models of human atrial and ventricular cells are given in Online Supplementary Material 1.1.

### Modeling state-dependent I_Na_ block

As in previous studies (Aguilar-Shardonofsky et al., 2012; Aguilar et al., 2015), I_Na_ block was simulated using a guarded receptor model with dynamical drug-channel interactions. This approach allows for investigations of the role of the specified parameters for selected I_Na_ blockers, and effects of combined I_Kur_ block on the atrial selectivity of Na^+^-channel block. The guarded receptor model considers the binding and unbinding kinetics of the drug to I_Na_ channels in a drug concentration-dependent manner. They can be described by first-order transition equations (Aguilar-Shardonofsky et al., 2012; Aguilar et al., 2015). It was also assumed that the drug predominantly binds to the activated and/or inactivated states of I_Na_. The blockade of I_Na_ is given by Aguilar-Shardonofsky et al. (2012) and Aguilar et al. (2015):

(1) $$\begin{array}{r} I_{\text{Na}}=g_{\text{Na}}\left(1-B_{A}-B_{I}\right)m^{3}hj\left(V_{m}-E_{Na}\right) \end{array}$$

(2) $$\begin{array}{r} \frac{dB_{A}}{dt}=K_{A}\left[D_{Na^{+}}\right]m^{3}hj\left(1-B_{A}-B_{I}\right)-L_{A}B_{A} \end{array}$$

(3) $$\begin{array}{r} \frac{dB_{I}}{dt}=K_{I}\left[D_{Na^{+}}\right]\left(1-h\right)\left(1-B_{A}-B_{I}\right)-L_{I}B_{I} \end{array}$$

where *g*_Na_ is the maximum conductance of I_Na_; *B*_*A*_ and *B*_*I*_ are the fractional blockade of activation and inactivation channels; *m* is the activation gate state variable, *h* and *j* are the inactivation gate state variables; *V*_m_ the transmembrane potential; *E*_*Na*_ the reversal potential of Na^+^; *K*_*A*_, *K*_*I*_ the binding constants and *L*_*A*_, *L*_*I*_ the unbinding constants; $\left[D_{Na^{+}}\right]\;$ is the concentration of a Na^+^-blocker. As in previous studies (Aguilar-Shardonofsky et al., 2012; Aguilar et al., 2015), a concentration of 60 μM was utilized unless otherwise stated; this concentration was chosen based on previous experimental and modeling studies (Zhu et al., 2006; Moreno et al., 2011; Aguilar-Shardonofsky et al., 2012; Aguilar et al., 2015); a parameter set (*K*_*A*_ = 100 ms^−1^· M^−1^, *K*_*I*_ = 100 ms^−1^· M^−1^, *L*_*A*_ = 1 ms^−1^, *L*_*I*_ = 0.01 ms^−1^) was first used to represent the kinetics of an I_Na_-selective blocker.

In our investigations of the AF-selectivity of I_Na_ block following AF-remodeling, the binding and unbinding constants of the I_Na_ blockers were varied to evaluate the dependence of I_Na_ block on these parameters, and whether an atrial-selective anti-AF action could be achieved in cAF-remodeled myoctes. The AF-selectivity of Na^+^-channel blockade was defined as the product of atrial-selectivity, rate-selectivity and block efficacy. With fractional block (*B*_f_) by Na^+^-channel blockers being measured as the relative reduction in the peak of I_Na_, the rate-selectivity was defined as the ratio of *B*_f_ measured in an atrial myocyte paced at 6 Hz to that paced at 1 Hz (Aguilar-Shardonofsky et al., 2012; Aguilar et al., 2015). Atrial-selectivity was used to determine the extent of atrial-ventricular difference in response to each drug. This was represented by the ratio of *B*_f_ observed from an atrial myocyte to that of a ventricular cell both paced at 1 Hz. In this study, we defined block efficacy (*E*) as:

(4) $$\begin{array}{r} E=\frac{1.0}{1.0+{\left(\frac{0.5}{B_{\text{f},6\text{Hz}}}\right)}^{4}} \end{array}$$

where B_f,6Hz_ is the fractional block of I_Na_ measured in an atrial cell paced at 6 Hz. Different from Aguilar et al. (2015), we introduced block efficacy to constrain the measure of AF-selectivity when the fractional block observed in a ventricular cell paced was minimal (and could result in a great atrial-selectivity), which otherwise could give a great value in AF-selectivity regardless of a small *B*_f,6Hz_.

To assess the dependence of the AF-selectivity of I_Na_ block on the drug action kinetics, the unbinding constants *L*_*A*_ and *L*_*I*_ were first varied over a parameter space from 10^−5^ to 10^0^ ms^−1^, while *K*_*A*_ and *K*_*I*_ were fixed (see Figure S6 of online Supplementary Materials for more details). The resultant unbinding constants were used in subsequent optimizations varying *K*_*A*_ and *K*_*I*_. The parameter space was {1, 10, 100, 500, 2,500, 10,000} for *K*_*A*_ and {1, 10, 100, 200, 500, 2,500} for *K*_*I*_. The parameter space fell into a likely range of I_Na_ blockers as summarized in Aguilar-Shardonofsky et al. (2012).

### Modeling effects of acacetin on atrial and ventricular electrophysiology

Acacetin was the chosen I_Kur_ blocker in the present study. To reveal the functional effects of (i) pure I_Kur_ block vs. (ii) the effects of combined K^+^ currents block by acacetin on human atrial electrophysiology, the actions of acacetin were modeled by considering its effects on (a) I_Kur_ only, and (b) all the respective K^+^ currents as detailed in Table 1. This approach allows for modeling the effects of the selective I_Kur_ block as well as uncovering the role of “additional” inhibitory effects of acacetin on other K^+^ currents.

**Table 1:** Concentration-dependent block of K^+^-currents by acacetin (Li et al., 2008; Wu et al., 2011).

	**I_Kur_**	**I_to_**	**I_Kr_**	**I_Ks_**
IC_50_ (μM)	3–3.2	9.3	32.4	81.4
Hill coefficient	0.8	0.9	0.9	0.8
Fractional inhibition at 3.2 μM	50%	28%	11%	7%

#### Modeling effect of acacetin on I_Kur_

Previous modeling studies have demonstrated the important role of the kinetic properties of drug actions in I_Kur_ block (Tsujimae et al., 2008; Almquist et al., 2010; Scholz et al., 2013). In addition, the pharmaceutical effects of acacetin on I_Kur_ are characterized by use- and rate-dependencies (Wu et al., 2011), which have also been observed in other I_Kur_ blockers (Pavri et al., 2012; Ford et al., 2016). Therefore, it was necessary to adopt a state-dependent block model (Brennan et al., 2009) for simulating the blockade of I_Kur_ by acacetin. Similar to our approach for modeling I_Na_ block, the binding and unbinding kinetics of a drug was described by a first-order transition equation, in contrast to simulating I_Kur_ block by reducing its conductance in Aguilar et al. (2015). Experimental studies revealed that acacetin binds to both the open and closed gates of K_V_1.5 (Wu et al., 2011). Therefore, following the guarded receptor formulas given in Equations (1–3), the formulation of inactivation-state binding and unbinding kinetics in I_Na_ block was modified to simulate the closed-state block of I_Kur_ by acacetin. The guarded receptor model of I_Kur_ block by acacetin is given by:

(5) $$\begin{array}{r} I_{\text{Kur}}=g_{\text{Kur}}\left(1-B_{O}-B_{C}\right)ai\left(V_{m}-E_{K}\right) \end{array}$$

(6) $$\begin{array}{r} \frac{dB_{O}}{dt}=K_{O}\exp\left(Z_{KO}\frac{V_{m}F}{RT}\right)\left[D_{K^{+}}\right]ai\left(1-B_{O}-B_{c}\right)\\ -L_{O}\exp\left(-Z_{LO}\frac{V_{m}F}{RT}\right)B_{O} \end{array}$$

(7) $$\begin{array}{r} \frac{dB_{c}}{dt}=K_{c}\exp\left(Z_{Kc}\frac{V_{m}F}{RT}\right)\left[D_{K^{+}}\right]\left(1-a\right)i\left(1-B_{O}-B_{c}\right)\\ -L_{c}\exp\left(-Z_{Lc}\frac{V_{m}F}{RT}\right)B_{c} \end{array}$$

where *g*_Kur_ is the conductance of I_Kur_; *B*_o_ and *B*_C_ are the fractional block on open and closed state variables, respectively; *a* and *i* are the activation and inactivation gate variables; *E*_*K*_ is the reversal potential of potassium; *F*, *R*, and *T* are the Faraday's constant, universal gas constant and temperature respectively. *K*_*O*_ and *K*_*c*_ are the binding constants; *L*_*O*_ and *L*_*c*_ are the unbinding constants; *Z*_*KO*_, *Z*_*LO*_, *Z*_*Kc*_ and *Z*_*Lc*_ are the drug charge parameters for the corresponding binding or unbinding processes; $\left[D_{K^{+}}\right]$ is the concentration of acacetin applied. The binding and unbinding parameters were obtained by fitting the model to the experimental data on the rate-dependent blockade of I_Kur_ by acacetin (Wu et al., 2011), as detailed in Online Supplementary Material 1.1.

**Figure 1:**
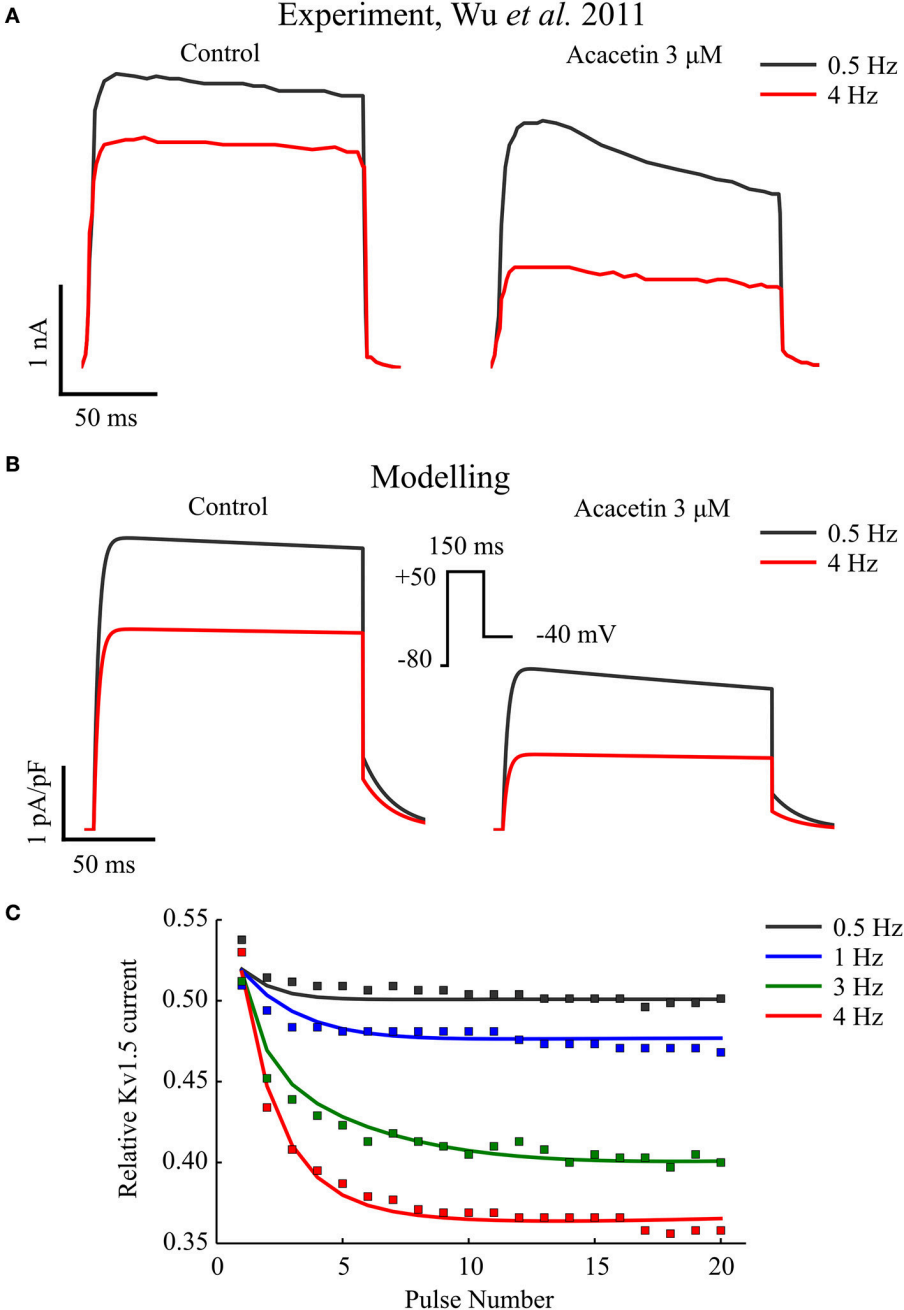
Frequency-dependent inhibition of I_Kur_ by acacetin. **(A)** Experimental and **(B)** simulated traces of K_V_1.5 channel current elicited from the 20th voltage step repeating at 0.5 and 4 Hz in control (left) and after exposure to 3 μM of acacetin (right). **(C)** Relative remaining I_Kur_ following application of acacetin at various frequencies plotted against the pulse number of the voltage step. The simulated data (lines) were compared with experimental values (squares). The relative fraction was obtained by normalizing the end-step current measured from each pulse following application of acacetin to that of control. Experimental data were digitalized from Wu et al. (2011).

Figure 1 shows a simulated frequency-dependent block of I_Kur_ by acacetin, and this is compared to the experimental data (Figures 1A,B). As shown, repeating the voltage command (Figure 1B, insert) at 0.5 Hz resulted in an approximately 50% blockade in this current after application of 3 μM acacetin. Increasing the voltage command rate to 4 Hz significantly increased the relative fractional block to approximately 63% (Figure 1C).

#### Modeling effect of acacetin on I_to_, I_Kr_, and I_Ks_

In addition to inhibiting I_Kur_ in the atria, acacetin potently blocks both I_to_ and I_K,ACh_, and also modulates I_Kr_ and I_Ks_, exhibiting multiple K^+^-current block. The parameters of Hill equations describing use-dependent inhibitions of these channels by acacetin are shown in Table 1. In the simulations, the effects of acacetin on these channels were modeled using a simple pore block model (Yuan et al., 2015). In the present study, we did not simulate the effects of acacetin on I_K,ACh_ inhibitions as the role of autonomic regulation on AF is beyond the scope of the study.

#### Simulations of the effects of acacetin on human ventricle

The effects of acacetin on human ventricular APs are unknown, although experimental data demonstrated that acacetin at 30 μM did not affect the heart rate and QT interval in isolated rabbit hearts (Li et al., 2008). In the present study, it was assumed that similar effects on the K^+^ currents (I_to_, I_Kr_, I_Ks_) in atrial myocytes could be extrapolated to the ventricular myocytes. We acknowledge that I_Kur_ is negligible in ventricles (Ravens and Wettwer, 2011), therefore in simulations of blocking I_Kur_ alone, the ventricular electrophysiology was not affected.

### Tissue models

The effects of acacetin and I_Na_ blockers on atrial and ventricular electrophysiology were further evaluated using tissue models. The monodomain equation (Clayton et al., 2011) was employed to simulate the excitation wave propagation in the myocardium. 1D models of human atrial strands were used to quantify the effects of channel blockers on atrial conduction velocity and APD restitution properties. Changes in ventricular depolarization and repolarization in response to these drugs were evaluated using a 1D model representing a transmural strand of ventricular tissue. In order to evaluate the antiarrhythmic effects of the channel blockers on re-entrant excitations in atria in the setting of cAF-induced remodeling, both idealized 2D models representing an isotropic slab of atrial tissue and an anatomically accurate 3D model of the human atria (Aslanidi et al., 2011; Colman et al., 2013, 2017; Whittaker et al., 2017) were employed to simulate the behavior of re-entrant excitations in atrial tissue. Pseudo-ECGs (pECGs) (Gima and Rudy, 2002; Baher et al., 2006) were computed as a measure of the excitation rates of in-tissue with sprial excitation waves. Detailed descriptions of these tissue models and pECGs are given in Online Supplementary Material 1.2, 1.3.

## Results

The updated Colman et al. human atrial model was first used to simulate effects cAF-induced remodeling on the action potential (AP) and calcium transient (CaT). Details are presented in Online Supplementary Material 2.1. The resultant changes in APD, APD restitution and CaT following cAF-induced remodeling as compared to those under the normal condition showed good agreement with previous experimental (Bosch et al., 1999; Osaka et al., 2000; Workman et al., 2001; Dobrev and Ravens, 2003; Voigt et al., 2012) and simulation studies (Zhang et al., 2005; Grandi et al., 2011; Colman et al., 2013, 2017; Wilhelms et al., 2013).

### Effects of application of acacetin on human atrial cells

**Figure 2:**
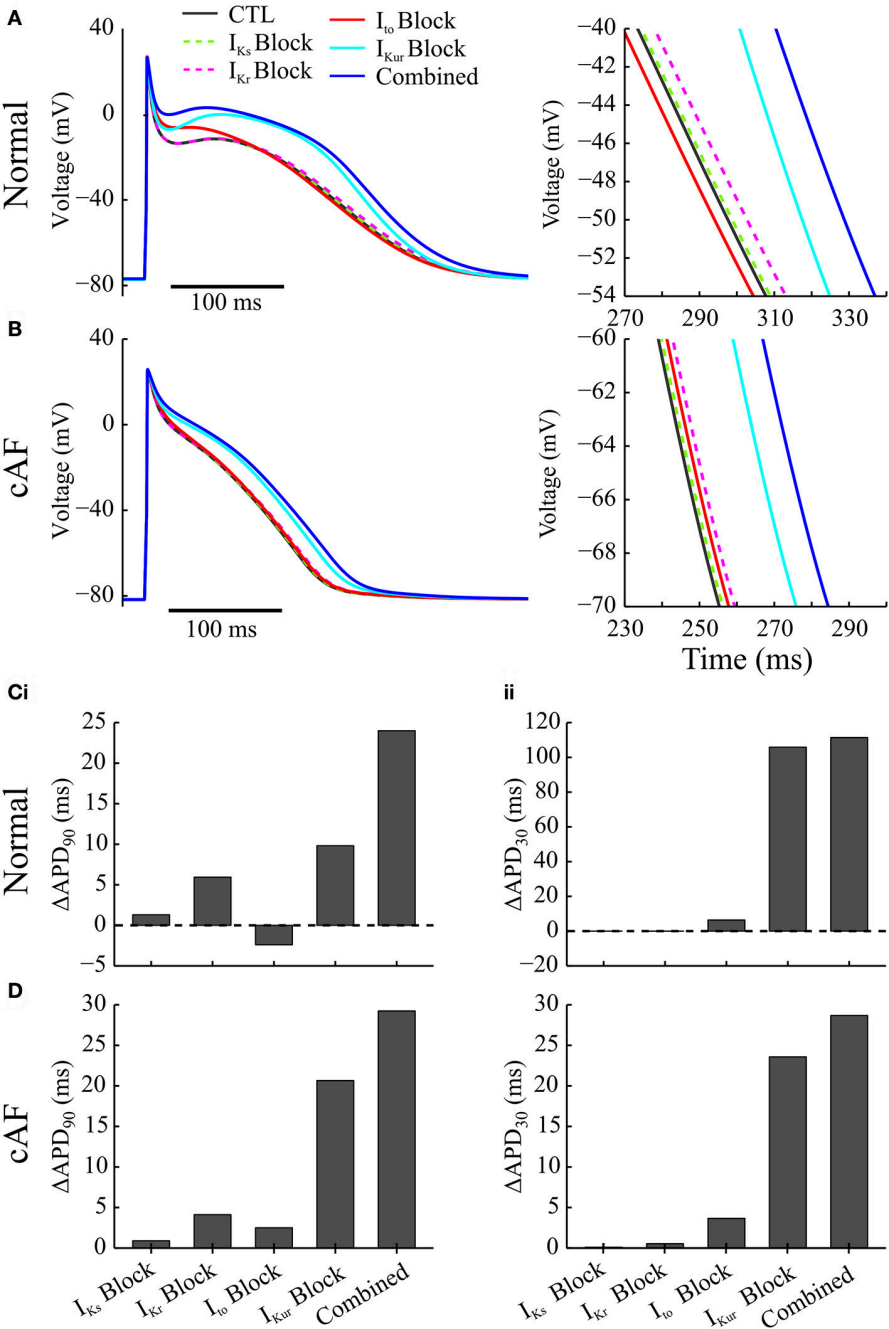
Effects of individual vs. combined block of K^+^-currents by acacetin (3.2 μM) on the human atrial AP in normal and cAF-remodeled myocytes paced at 1 Hz. **(A,B)** Effects on the atrial AP in **(A)** normal and **(B)** cAF-remodeled myocytes; a zoomed-in view for the traces of AP during phase-3 is plotted to the right. **(C,D)** Alterations in the **(i)** APD_90_ and **(ii)** APD_30_ by the simulated block obtained from atrial cells in **(C)** normal and **(D)** cAF-remodeled myocytes.

To reveal the roles of inhibition of individual channels by acacetin in modulating cellular AP by acacetin, both the individual and combined block of I_to_, I_Kr_, I_Kur_, and I_Ks_ by acacetin (3.2 μM) in simulated SR at cycle length 1,000 ms without (normal) or with cAF-related electrical remodeling were simulated. Figures 2A,B illustrates the effects of individual and combined K^+^-channel block by acacetin on AP waveform. The alterations to APD relative to the control are summarized in Figures 2C,D.

In the absence of electrical remodeling, in normal myocytes at a cycle length of 1,000 ms, simulated I_Ks_ or I_Kr_ block by acacetin (3.2 μM) presented no significant alterations to the atrial AP: although the atrial repolarization was delayed by 1.3 and 5.9 ms, respectively, the plateau phase was not affected, which is consistent with the minimal potency of acacetin on these channels (Table 1). Similar effects were also obtained from our simulated I_Kr_ and I_Ks_ block by the compound in the cAF-remodeled atrial cells.

Selective block of I_to_ (alone) by acacetin elevated the atrial plateau potential in both normal and cAF-remodeled myocytes paced at 1 Hz, and this led to modest prolongations in APD_30_ (by 6.4 and 3.6 ms for normal and AF-remodeling myocytes, respectively). The changes in APD_90_ due to I_to_ block varied between the two conditions: under normal conditions the atrial APD_90_ was shortened by 2.4 ms, whereas it was prolonged by 3.7 ms following cAF-remodeling at a stimulus rate of 1 Hz.

In contrast, blocking I_Kur_ alone by acacetin resulted in a pronounced alteration to the shape and duration of the AP in both normal and cAF-remodeled myocytes. The inhibition in I_Kur_ significantly elevated the plateau potential of atrial AP (by 7.1 and 5.7 mV in normal and cAF-remodeled myocytes, respectively), and this was accompanied by marked prolongations in APD_30_ (by 105.9 and 23.6 ms in normal and cAF-remodeled myocytes, respectively). The prolongation in APD_90_ induced by the I_Kur_ block was 9.8 ms for normal atrial cells, and was more pronounced (23.6 ms) in cAF-remodeled myocytes, despite that I_Kur_ was down-regulated by cAF-remodeling.

We note that combined effects of acacetin (3.2 μM) on multiple K^+^-currents produced greater alterations to the AP than those of any individual blocking effect. We have quantified effects produced by the combined block and compared it with the sum of the changes seen in each individual block. Synergistic effects were observed in the changes in APD_90_, represented by a further prolongation of 9.3 and 1.1 ms in APD_90_ in normal and cAF-remodeled myocytes, respectively. Additionally, the effects of I_Kur_ block dominated the AP-modulation by the compound, which is consistent with the high potency of acacetin on the channel (Table 1).

### Effects of sodium blocker and acacetin on cAF-remodeled atrial myocytes and ventricular cells

#### Effects on single myocyte AP and I_Na_

**Figure 3:**
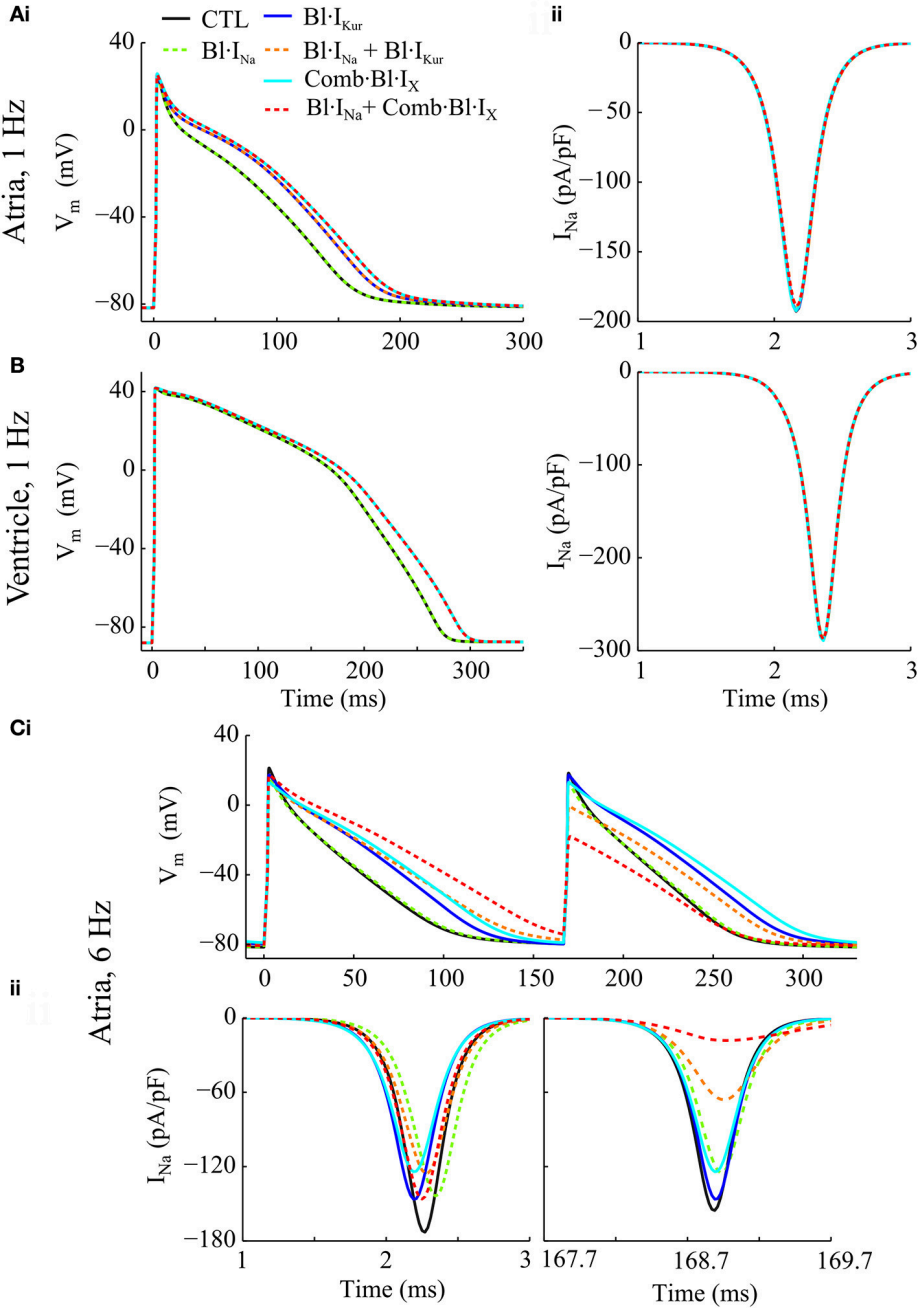
Simulated AP and I_Na_ traces of cAF-remodeled atrial myocytes and ventricular cells in response to Na^+^- and K^+^- block regimens. **(Ai)** APs from cAF-remodeled atrial myocytes paced at 1 Hz; **(Aii)** Time courses of corresponding I_Na_ during the upstroke phase. **(B)** Simulated time courses of AP and I_Na_ of a ventricular cell paced at 1 Hz. **(C)** Illustration of **(i)** APs and **(ii)** the corresponding time courses of I_Na_ of a cAF-remodeled myocyte paced at 6 Hz. In these simulations, rate constants for I_Na_ blocker were: *K*_A_ = 100 ms^−1^· M^−1^, *K*_I_ = 100 ms^−1^· M^−1^, *I*_A_ = 1 ms^−1^, *I*_I_ = 0.01 ms^−1^.

**Figure 4:**
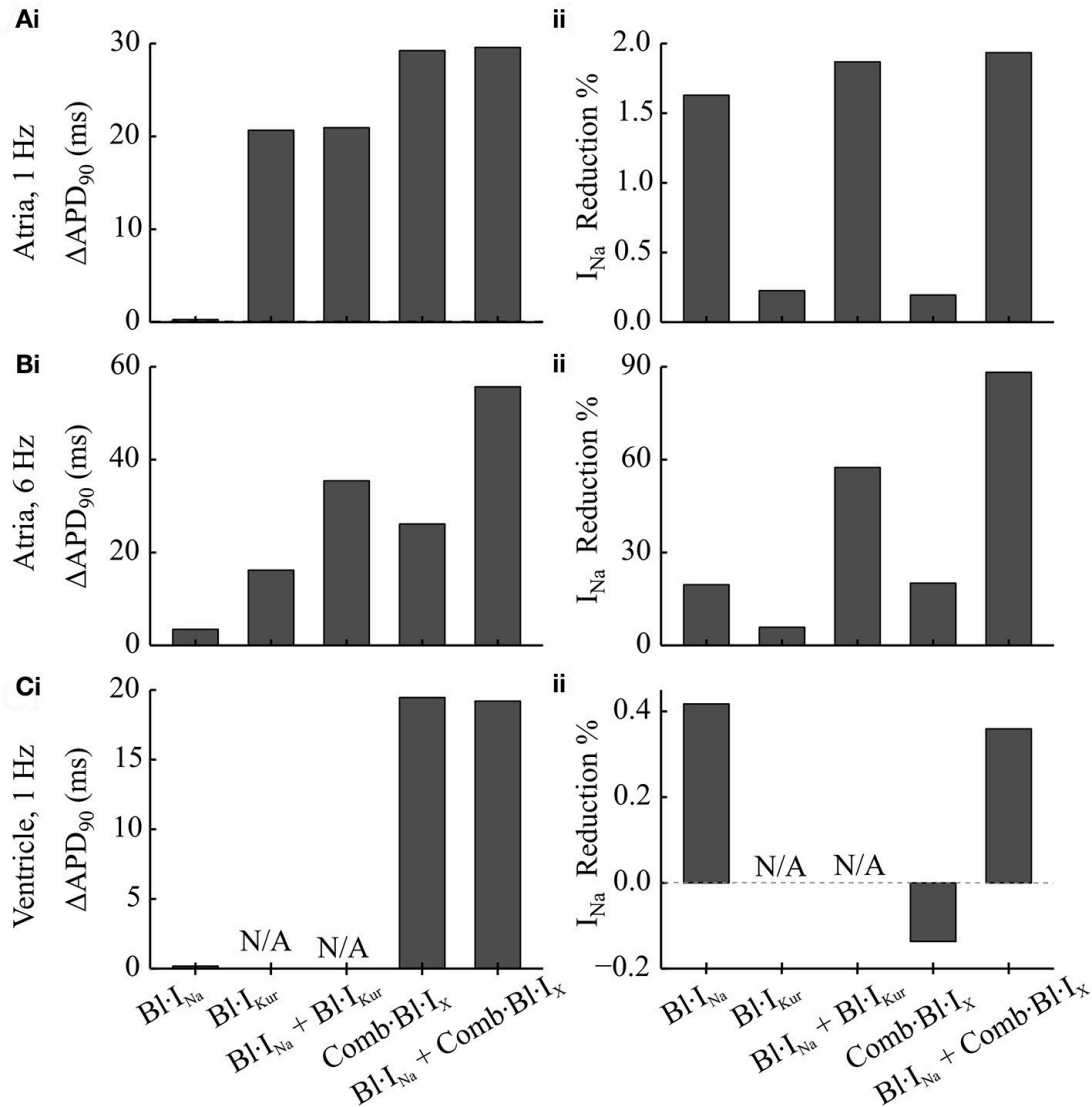
Simulated changes in the APD and peak I_Na_ following the applications of Na^+^- and K^+^- block in comparison to the drug-free condition in cAF-remodeled atrial myocytes or ventricular cells. **(A,B)** Changes in **(i)** APD and **(ii)** peak I_Na_ measured from a cAF-remodeled atrial myocyte paced at **(A)** 1 Hz and **(B)** 6 Hz. In the presence of alternans, the changes in APD were quantified by comparing the corresponding longer APs. The fractional reductions in peak I_Na_ were calculated from the I_Na_ of the corresponding shorter APs. **(C)** Changes in **(i)** APD and **(ii)** peak I_Na_ measured from an *in silico* ventricular myocyte paced at 1 Hz.

Individual and combined effects of Na^+^-block (indicated by Bl·I_Na_) and K^+^-block by acacetin (3.2 μM) on human atrial electrophysiology after cAF-remodeling were simulated to assess any anti-AF benefits. Effects of acacetin (representing K^+^-block) were simulated in different settings: (i) I_Kur_ block alone (denoted by Bl·I_Kur_) and (ii) combined block of all K^+^-currents in Table 1 (denoted by Comb·Bl·I_X_). In addition, the effects of Na^+^- and K^+^- block on human ventricular myocytes were also studied to assess the atrial-selectivity of the block. The results are shown in Figures 3A–C and quantitative measurements are shown in Figures 4A–C.

For atrial myocytes paced at 1 Hz, Bl·I_Kur_, and Comb·Bl·I_X_ prolonged atrial APD (Figure 4Ai and as presented in Effects of Application of Acacetin on Human Atrial Cells) whilst their effects on peak I_Na_ were minimal (reducing peak I_Na_ by less than 0.3%). Bl·I_Na_ alone slightly reduced the peak I_Na_ by 1.63% without affecting the APD. The fractional inhibition in I_Na_ by I_Na_-block was slightly increased by the addition of Bl·I_Kur_ or Comb·Bl·I_X_ (Figure 4Aii). In the ventricles, the simulated application of acacetin induced a prolongation of 19.5 ms in APD_90_ compared with that in control (drug-free) condition (Figures 3B, 4Ci). Bl·I_Na_ alone showed a negligible inhibitory effect on the ventricular I_Na_ (by 0.42%), which was also not affected by combining Bl·I_Na_ and Comb·Bl·I_X_ (Figure 4Cii).

In atrial myocytes paced at 6 Hz, AP alternans were observed under the drug-free condition (Figure 3Ci): the APD varied between 100.1 and 88.1 ms. In the presence of AP alternans, the changes in APD by the Na^+^- and K^+^- blockers were quantified by comparing the corresponding big APs at baseline and after drug actions. These values were selected based on the characteristics of AP (APD prolongations seen in the long AP, and reduced I_Na_ for the short AP) that may be anti-arrhythmic. The fractional reductions in peak I_Na_ were calculated from the I_Na_ associated with the shorter APs. The results showed that applying Bl·I_Kur_ or Comb·Bl·I_X_ alone both abolished the AP alternans while prolonging the APD to 116.3 and 126.3 ms and reducing the peak I_Na_ by 5.9 and 20.1%, respectively. The application of Bl·I_Na_ alone produced a minor APD prolongation (3.5 ms) and a reduction of 16.2% in peak I_Na_. Combining block of I_Na_ with Bl·I_Kur_ or Comb·Bl·I_X_ promoted the genesis of AP alternans, resulting in substantial prolongations in the APD of the big APs (by 35.4 ms for Bl·I_Kur_ + Bl·I_Na_, and 55.6 ms for Comb·Bl·I_X_ + Bl·I_Na_) and dramatic decreases in the peak I_Na_ (by 57.5% for Bl·I_Kur_ + Bl·I_Na_ and 88.2% for Comb·Bl·I_X_ + Bl·I_Na_) in the corresponding small APs. These results suggest that the combined block of Bl·I_Na_ and Comb·Bl·I_X_/Bl·I_Kur_ exhibited synergistic antiarrhythmic effects manifested by prolongation in APD and reduction in peak I_Na_. However, an increased susceptibility to AP alternans was observed at a fast pacing rate of 6 Hz, which may be potentially proarrhythmic at fast heart rates.

#### Effects on steady-state restitutions of APD and conduction velocity

Steady-state APD restitutions of cAF-remodeled human atria were simulated at both the cellular and tissue levels. In single myocyte simulations (Figure S5 in Online Supplementary Material 2.2), APD was prolonged over the entire range of simulated basic cycle lengths (BCL) for Bl·I_Kur_ and Comb·Bl·I_X_ as compared to the control (drug-free) conditions. The reduction in peak I_Na_ in Bl·I_Na_ was rate-dependent and significantly greater at fast pacing rates. K^+^-block alone (Bl·I_Kur_ or Comb·Bl·I_X_) slightly shifted the rate-dependence of peak I_Na_ to larger BCLs. In comparison to the effects of individual current block scenarios, synergistic reductions in peak I_Na_ were observed following combined blocks of Bl·I_Na_ with Bl·I_Kur_ or Comb·Bl·I_X_ over a wide range of BCLs. As compared to the drug-free conditions, AP alternans were observed at greater BCLs after K^+^-block, and this was further increased by combined Na^+^- and K^+^-block (Figure S5 in Online Supplementary Material 2.2).

**Figure 5:**
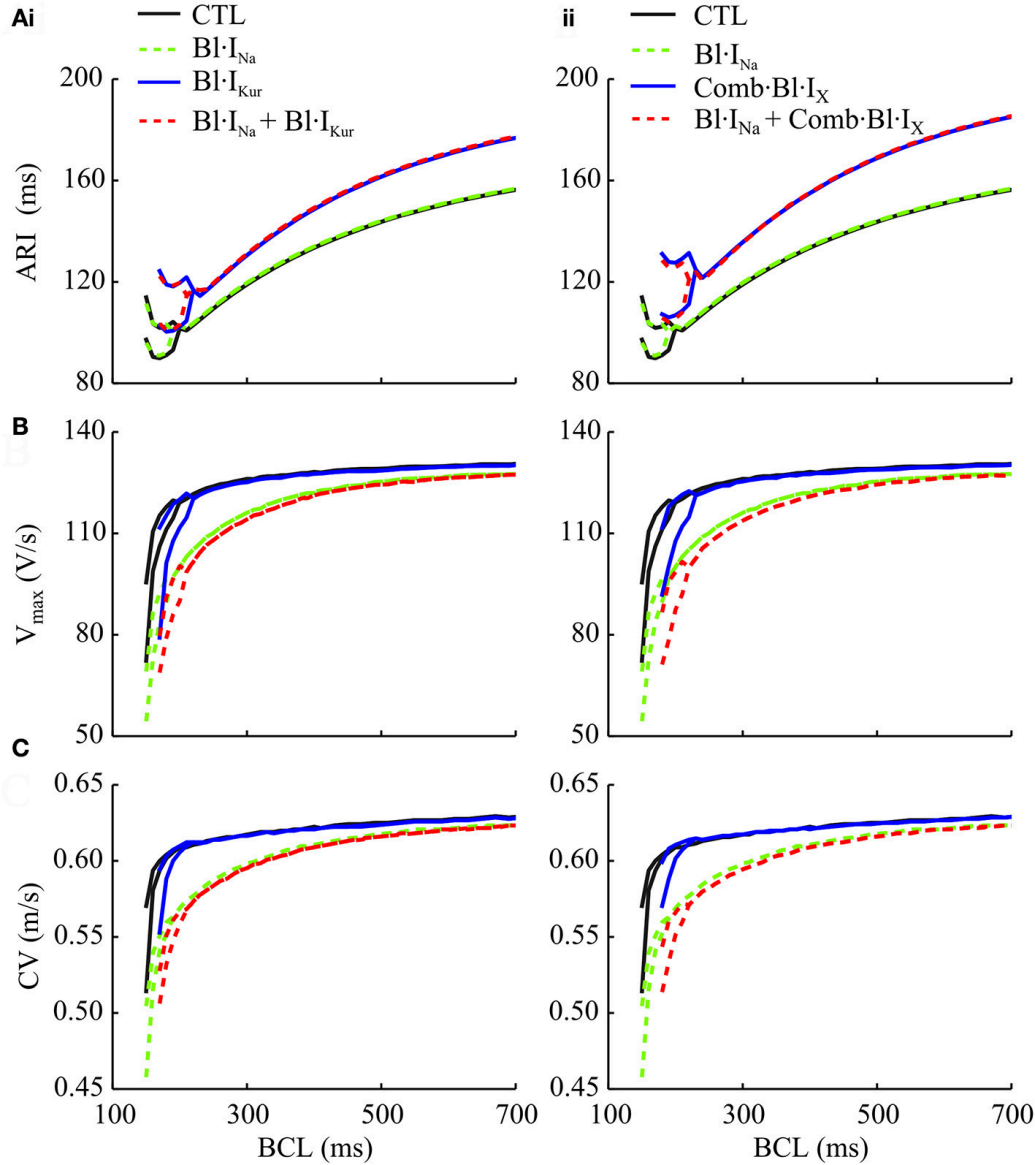
Simulated activation-recovery intervals (ARI) **(A)**, V_max_ **(B)**, and CV (conduction velocity) **(C)** measured in 1D cAF-remodeled atrial strand models as a function of BCL for control (drug-free), individual and combined Na^+^- and **(i)** I_Kur_ block and **(ii)** action of acacetin. ARI values were measured as the interval between the time at which the AP depolarizes to −20 mV and the time it reaches a 90% repolarization.

Using a 1D model of atrial strands, the atrial APD and conduction velocity (CV) restitutions, as well as the rate-adaptation of in-tissue upstroke velocity (V_max_), were evaluated (Figure 5). The atrial activation-recovery interval (ARI) was not affected by Bl·I_Na_ alone, whereas it was substantially lengthened by K^+^-block as compared to control (Figure 5A). Applying K^+^-block alone also shifted the CV and V_max_ restitution curves rightwards (i.e., to higher BCLs) (Figures 5B,C). These rate-adaptations of V_max_ and CV were progressively enhanced by Bl·I_Na_ and the combined block over a wide range of BCLs. Synergistically enhanced rate-dependent reductions in V_max_ and CV were observed in response to the combined blocks. Furthermore, K^+^-block increased the critical BCLs for conduction block as compared to the drug-free condition (Figure 5C).

### Effects of combined Na^+^- and K^+^- block on the AF-selectivity of I_Na_ block

AF-selectivity of Na^+^ blockers in cAF-remodeled hearts was examined by varying the drug binding and unbinding constants over wide parameters spaces to provide information concerning drug-Na^+^-channel interactions for various drug candidates. This was done by independently changing *L*_*A*_ and *L*_*I*_ for fixed {*K*_*A*_, *K*_*I*_} (Online Supplementary Material 2.3); and then varying *K*_*A*_ and *K*_*I*_ for fixed {*L*_*A*_, *L*_*I*_}. In this way we obtained the maximum AF-selectivity over the parameter space of drug binding and unbinding kinetics. Simulations with varied *K*_*A*_ and *K*_*I*_ were repeated for Bl·I_Na_ + Bl·I_Kur_ and Bl·I_Na_ + Comb·Bl·I_X_.

**Figure 6:**
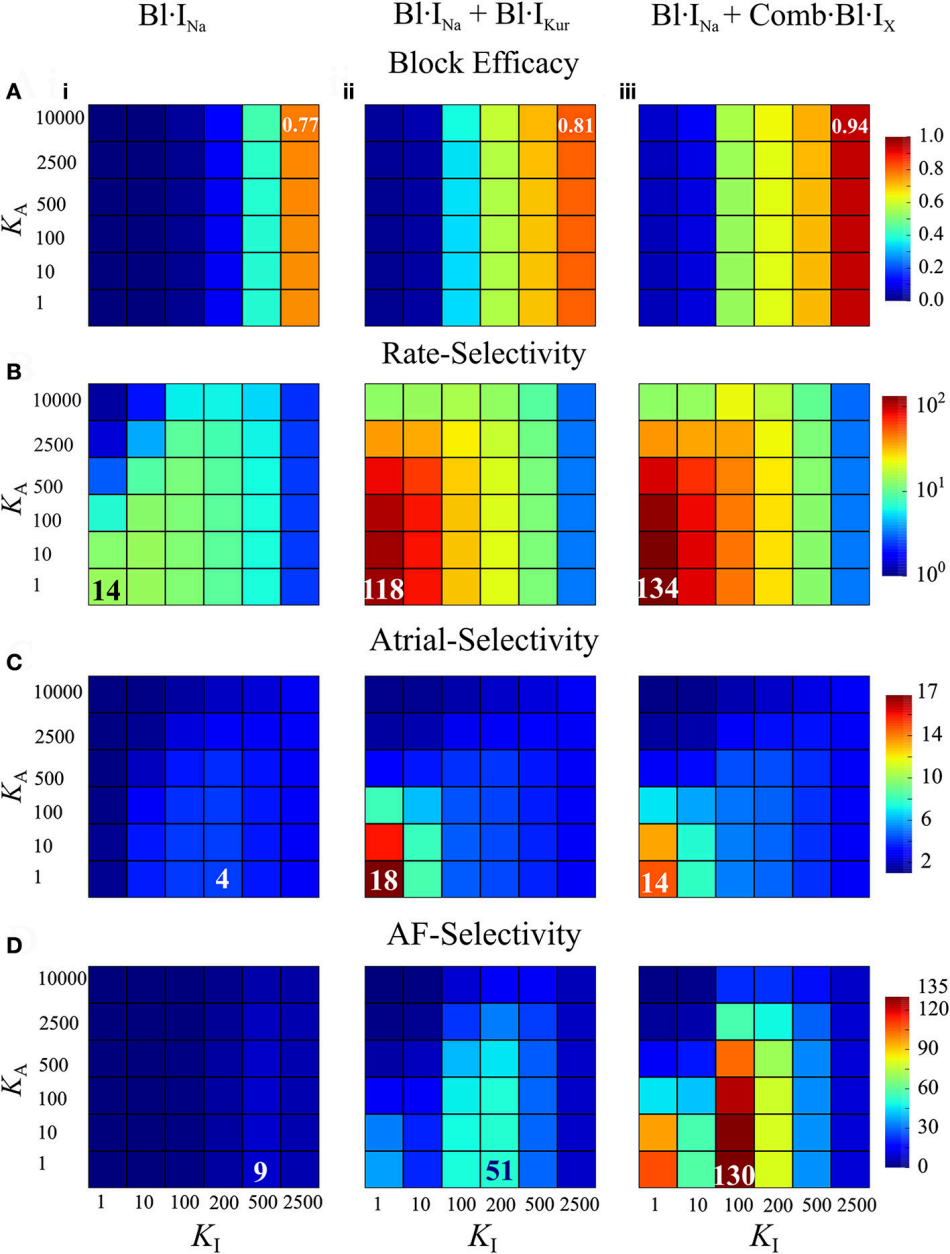
Block efficacy, and the rate-, atrial-, and AF-selectivity as a function of the open- and inactivated- state binding rates (*K*_*A*_, *K*_*I*_) for Na^+^-block or combined Na^+^- and K^+^-block computed from cAF-remodeled atrial myocytes or ventricular myocytes. **(A)** Block efficacy; **(B)** rate-selectivity; **(C)** atrial-selectivity, and **(D)** AF-selectivity. In each column shown are data from Bl·INa **(i)**, combined Bl·INa and Bl·IKur **(ii)**, and combined Bl·INa and Comb·Bl·IX **(iii)**. $L_{A}=1\text{ m}{\text{s}}^{-1},L_{I}=0.01\text{ m}{\text{s}}^{-1}$. Unit of *K*_*A*_, *K*_*I*_: ms^−1^·M^−1^.

Figures 6A–D illustrates the block efficacy (defined in Equation 4), rate-selectivity, atrial-selectivity and the resultant AF-selectivity for Bl·I_Na_ alone and the combined block as a function of *K*_*A*_ and *K*_*I*_. For Bl·I_Na_ alone, the block efficacy increased with increase of *K*_I_, whereas the rate-selectivity was reduced by increasing *K*_*A*_ or *K*_*I*_. The AF-selectivity reached a maximum value of 9 at *K*_*A*_ = 1 and $K_{I}=500\;{\text{ms}}^{-1}\cdot {\text{M}}^{-1}$. The combined blocks achieved significantly greater AF-selectivity than Bl·I_Na_ alone: the maximum attainable AF-selectivity was increased by nearly 6-fold for Bl·I_Kur_ + Bl·I_Na_ and more than 14-fold for Comb·Bl·I_X_ + Bl·I_Na_ as compared to Bl·I_Na_ alone (Figure 6C). These dramatic increases were attributed to the significantly greater values in all metrics contributing to the AF-selectivity. The maximal block efficacy achieved by Bl·I_Na_ alone was 0.77, and was increased to 0.81 and 0.94 for Bl·I_Kur_ + Bl·I_Na_ and Comb·Bl·I_X_ + Bl·I_Na_, respectively. A more appreciable increase in the maximal rate-selectivity was observed by the combined blocks as compared to Bl·I_Na_ alone (8-fold for Bl·I_Kur_ + Bl·I_Na_ and nearly 10-fold for Comb·Bl·I_X_ + Bl·I_Na_). Additionally, the atrial-selectivity was also increased by the combined block, although to a lesser extent. Bl·I_Kur_ + Bl·I_Na_ exhibited a greater atrial-selectivity than that of Comb·Bl·I_X_ + Bl·I_Na_ since Bl·I_Kur_ was assumed to have no effect on the ventricles.

Furthermore, these simulations revealed that the block efficacy, rate-selectivity and atrial-selectivity were strongly dependent on the inactivation state binding rate *K*_*I*_. These measures were also dependent on the open-state binding kinetics *K*_*A*_, but to a much lesser extent. The block efficacy was mainly determined by *K*_*I*_: an increase in *K*_*I*_ led to a significant increase in the block efficacy. In combined block, the maximal rate- and atrial-selectivity were observed for $K_{A}=K_{I}=1\text{ m}{\text{s}}^{-1}\cdot {\text{M}}^{-1}$ and increases in *K*_*I*_ resulted in substantial reductions in the rate- and atrial-selectivity. In Bl·I_Na_ alone, the parameter set $K_{A}=1\text{ m}{\text{s}}^{-1}\cdot {\text{M}}^{-1},\;K_{I}=200\text{ m}{\text{s}}^{-1}\cdot {\text{M}}^{-1}$ produced a maximal value in atrial-selectivity. Collectively, the optimal *K*_*I*_ that maximized AF-selectivity was 200 ms^−1^·M^−1^ for Bl·I_Na_ and Bl·I_Kur_ + Bl·I_Na_, and smaller (100 ms^−1^·M^−1^) for Comb·Bl·I_X_ + Bl·I_Na_. The optimal $K_{A}=1\text{ m}{\text{s}}^{-1}\cdot {\text{M}}^{-1}$ was seen for all conditions. Increasing *K*_*A*_ consistently resulted in a smaller rate- and atrial-selectivity and therefore reduced AF-selectivity. These results suggest that the inactivation-state binding rate might be a more favorable targeting parameter than the open-state binding kinetics in optimizing AF-selectivity of Na^+^-blockers.

### Effects of I_Na_ and I_Kur_ block on spiral excitation events in cAF-remodeled atria

#### Two-dimensional simulations

**Figure 7:**
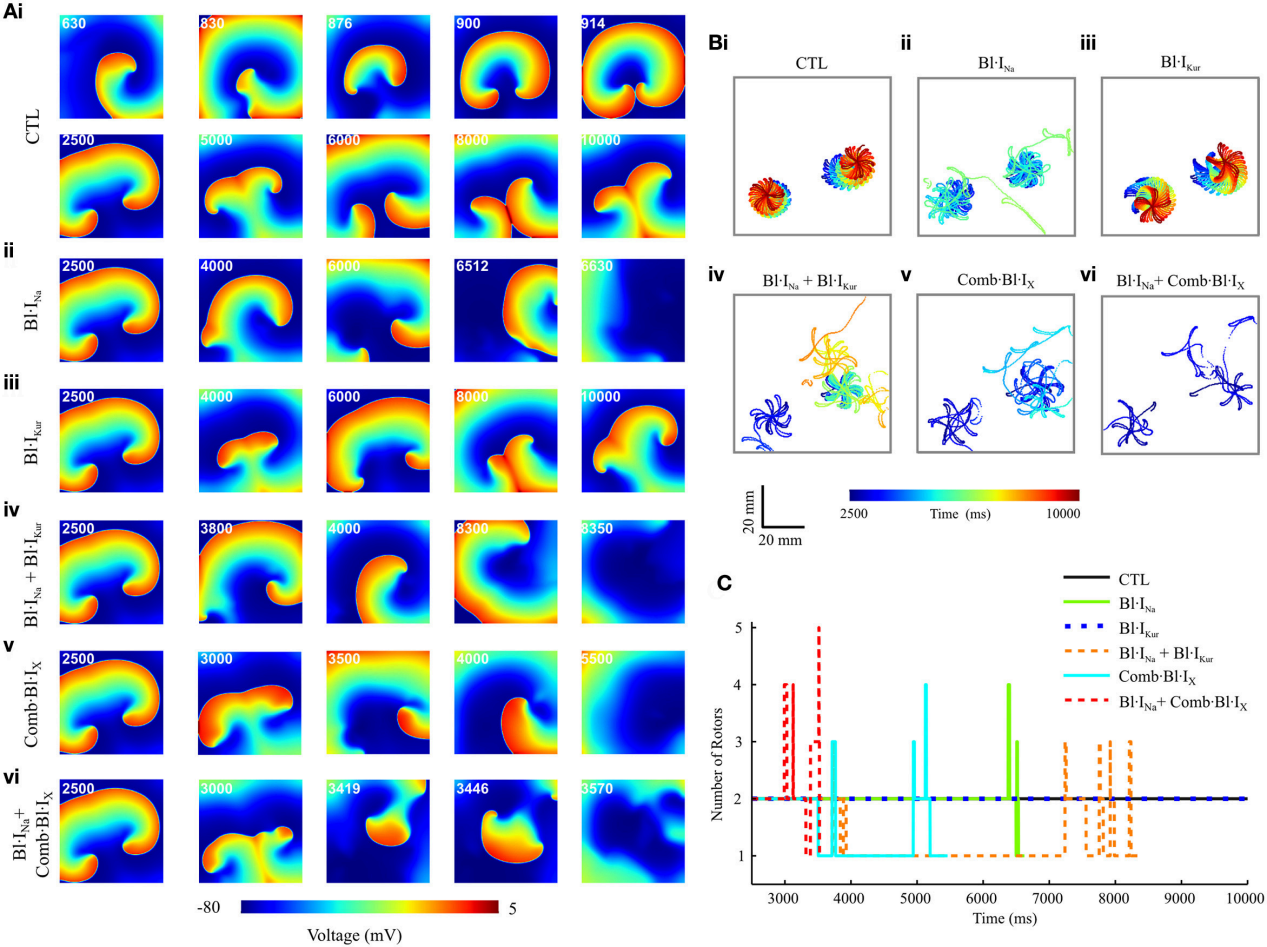
Snapshots of simulated re-entrant excitation events, tip trajectories of re-entrant waves and the number of rotor in a 2D model of cAF-remodeled atrial tissue slab in drug-free condition or after applying the drugs. **(A)** Snapshots of simulated re-entrant excitation events; The time sequence (ms) is indicated at the top left corner of each screenshot. **(B)** Tip trajectories of re-entrant waves. **(C)** Temporal evolution of total number of rotors represented by the number of spiral wave tips in tissue. In both **(A,B)**, **(i)** Drug-free (CTL) condition, **(ii)** Bl·I_Na_ alone, **(iii)** Bl·I_Kur_ alone, **(iv)** Combined Bl·I_Na_ and Bl·I_Kur_, **(v)** Applying Comb·Bl·I_X_ alone, and **(vi)** Combined Bl·I_Na_ and Comb·Bl·I_X_. Rate constants for I_Na_ block: $K_{A}=1\text{ m}{\text{s}}^{-1}\cdot {\text{M}}^{-1},K_{I}=100\text{ m}{\text{s}}^{-1}\cdot {\text{M}}^{-1},L_{A}=1\text{ m}{\text{s}}^{-1},\;L_{I}=0.01\text{ m}{\text{s}}^{-1}$.

Using the cross-shock protocol, spiral waves were initiated in a 2D model representing a tissue slab of cAF-remodeled human atria. For each condition, a 10-s episode of electrical activity was simulated. Representative snapshots of the re-entrant waves in control (drug-free) and following application of drugs are presented in Figure 7A. The trajectories of the tips of re-entrant rotors under these conditions were traced and are shown in Figure 7B. The number of rotors during the time course of wave evolution was also measured (Figure 7C). The simulated pseudo-ECGs, membrane potential traces extracted from a representative myocyte and the corresponding fractional block of I_Na_ and I_Kur_ are detailed in Online Supplementary Material 2.4.

Under the control (drug-free) condition, a single rotor was formed at approximately *t* = 630 ms; this broke into two spiral waves at *t* = 830 ms. These two rotors were stably anchored with star-shaped tip trajectories at the bottom half of the slab and persisted throughout the rest of the simulated 10-s episode (Figures 7Ai,Bi,C). In simulating drug actions, each drug was applied at *t* = 2,500 ms. For Bl·I_Na_ the dual rotors progressively became unstable, and the tips of the spiral waves meandered out of the tissue at approximately *t* = 6,000 ms, leading to self-termination of the re-entrant waves (Figures 7Aii,Bii,C). The dual rotors persisted throughout the period of the simulation after applying Bl·I_Kur_ alone (Figures 7Aiii,Biii,C). For Bl·I_Na_ + Bl·I_Kur_, the rotor at the bottom left corner of the slab became unstable and meandered out of the tissue at approximately *t* = 3,800 ms, whereas the trajectory of the second rotor was confined to a small tissue area until *t* = 7,000 ms and then gradually became chaotic, forming up to 3 transient rotors that self-terminated at *t* = 8,334 ms (Figures 7Aiv,Biv,C). A similar but more marked effect was seen in the simulations that addressed the aggregate effects of acacetin: the bottom left rotor quickly meandered out of the tissue at *t* = 3,510 ms whilst the tip trajectory of the other rotor became chaotic and terminated at *t* = 5,439 ms (Figures 7Av,Bv,C). We note that the combined Bl·I_Na_ and Comb·Bl·I_X_ exerted the strongest potency in terminating re-entrant excitations in these simulations: the two rotors transiently turned unstable and chaotic and self-terminated at *t* = 3,555 ms, with up to 5 rotors during the excitation in the slab (Figures 7Avi,Bvi,C).

**Figure 8:**
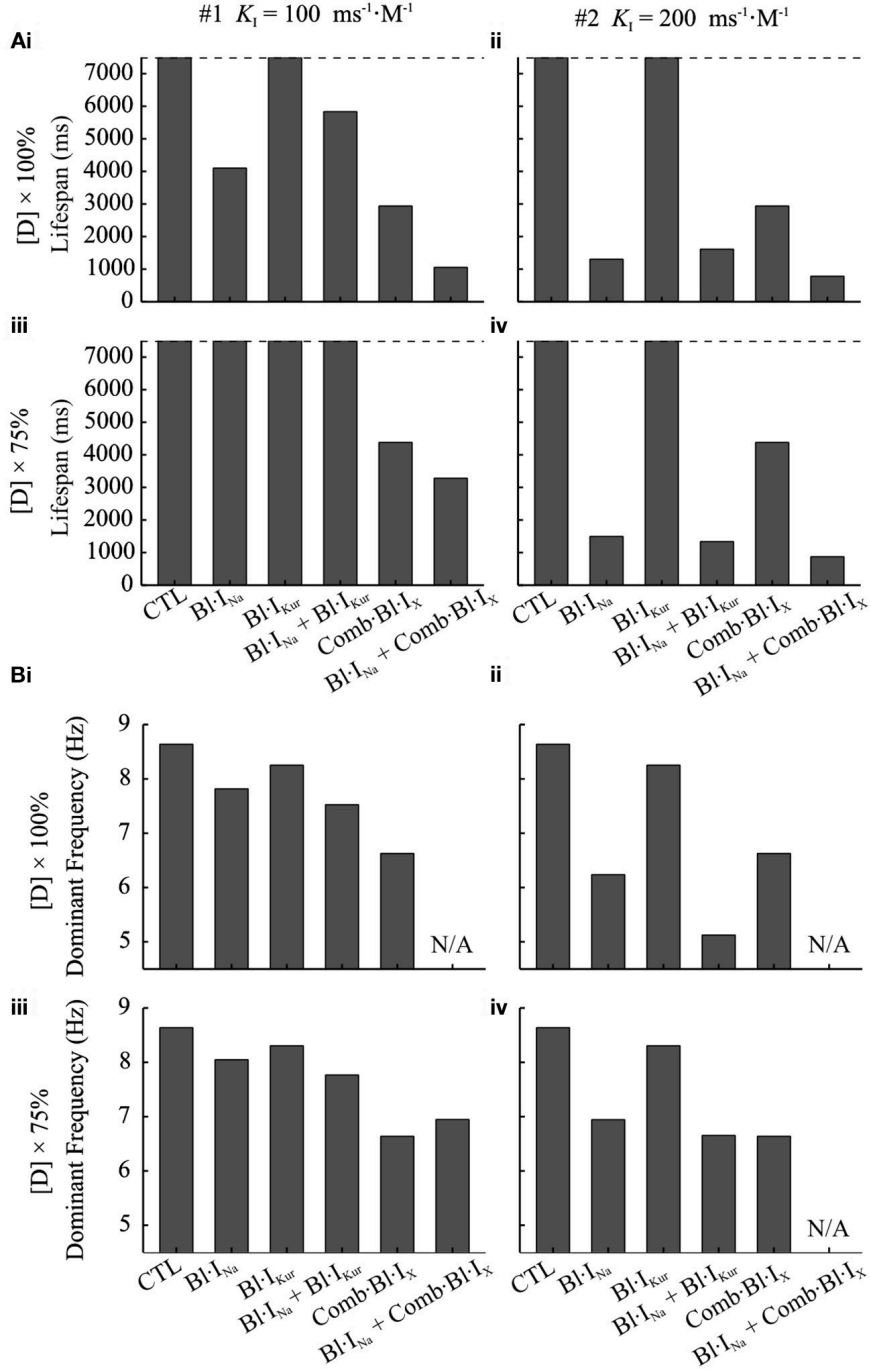
Dominant frequency and lifespan of re-entrant waves in 2D simulations using the cAF-remodeled atrial tissue model in the drug-free condition or after applying drugs. **(A)** Lifespan of the spiral waves. **(B)** DF of spiral waves. The results of K^+^-block alone are shown in both left and right panels for the purpose of comparison. In the left column **(i,iii)** of **(A,B)**, Bl·I_Na_ was simulated with $K_{I}=100\text{ m}{\text{s}}^{-1}\cdot {\text{M}}^{-1}$; in the right column **(ii,iv)**, $K_{I}=200\text{ m}{\text{s}}^{-1}\cdot {\text{M}}^{-1}$. In the top panels **(i,ii)** of **(A,B)**, $\left[D_{K^{+}}\right]=3.2\mu$M, $\left[D_{Na^{+}}\right]=60\mu$M; in the bottom panels **(iii,iv)**, $\left[D_{K^{+}}\right]=\;2.4\mu$M, $\left[D_{Na^{+}}\right]=\;45\;\mu$M. In all panels, $K_{A}=1\;{\text{ms}}^{-1}\cdot {\text{M}}^{-1},$ $L_{A}=1\text{ m}{\text{s}}^{-1},\;L_{I}=0.01\text{ m}{\text{s}}^{-1}$.

The anti-arrhythmic benefits of combined Na^+^- and K^+^- block were clearly revealed by additional simulations assuming the use of a different Na^+^-blocker ($K_{A}=1\text{ m}{\text{s}}^{-1}\cdot {\text{M}}^{-1},K_{I}=200\text{ m}{\text{s}}^{-1}\cdot {\text{M}}^{-1},L_{A}=1\text{ m}{\text{s}}^{-1},L_{I}=0.01\text{ m}{\text{s}}^{-1}$) and at a reduced dose (75%) of both Na^+^-blocker and acacetin. The life span of re-entrant excitations was measured and shown in Figure 8A. Also, the pECG was computed and the segment from *t* = 3,000 ms to 500 ms before the termination of re-entries (or the end of the simulation if the rotor sustained) was analyzed using the Fast Fourier Transform to obtain the dominant frequency (DF) of the re-entrant excitations, which is illustrated in Figure 8B.

At the simulated doses of acacetin, applying Bl·I_Kur_ alone did not lead to termination of re-entrant waves within the duration of the simulation (7,500 ms after T_Drug_), whereas the rotors were terminated in the simulations for Comb·Bl·I_X_ at both doses (Figure 8Ai–iv), thus demonstrating enhanced anti-AF benefits of combined K^+^-channel block. For the simulated Bl·I_Na_ alone, the Na^+^-blocker with $K_{I}=100\text{ m}{\text{s}}^{-1}\cdot {\text{M}}^{-1}$ led to termination of AF at the control dose (lifespan of 4,102 ms) but not at the reduced dose; increasing *K*_*I*_ of the Na^+^-blocker to 200 ms^−1^·M^−1^ resulted in a reduced lifespan (1,305 and 1,459 ms for $\left[D_{Na^{+}}\right]=60$ and 45 μM, respectively). The lifespan for the combined Bl·I_Na_ + Comb·Bl·I_X_ was consistently shorter than that of any individual applications of Bl·I_Na_ or Comb·Bl·I_X_ alone in all cases. A similar augmented anti-arrhythmic effect (shown as shortened lifespan of re-entry) was also observed for the combined Bl·I_Na_ + Bl·I_Kur_ for $\left[D_{Na^{+}}\right]$ = 45 μM and $K_{I}=200\text{ m}{\text{s}}^{-1}\cdot {\text{M}}^{-1}$ (Figure 8Aiv) but not for the rest of the cases.

A consistent decrease in the DF was observed in the drug-modulated re-entrant excitations as compared to those in the drug-free condition (Figure 8Bi–iv). In the drug-free condition, the DF extracted from the pECG was 8.63 Hz, which is within the range of similar clinical data (Jarman et al., 2012). Applying Na^+^- or K^+^- block individually resulted in slowing of the rate of the rotors, and this was dependent on the concentrations and parameters of the blockers. For Bl·I_Kur_ the DF was 8.25 Hz with the control dose and 8.30 Hz for the reduced dose. In the simulations with Comb·Bl·I_X_, the DF was 6.63 Hz and was not affected by the 25% reduction in the dose of the compound. For Bl·I_Na_ alone the DF was 7.81 Hz and substantially smaller (6.24 Hz) for Na^+^-blockers of $K_{I}=100\text{ m}{\text{s}}^{-1}\cdot {\text{M}}^{-1}$ and $K_{I}=200\text{ m}{\text{s}}^{-1}\cdot {\text{M}}^{-1}$, respectively. An enhanced deceleration of the rotors was observed for Bl·I_Na_ + Bl·I_Kur_ in all cases. The DF for simulations with Comb·Bl·I_X_ + Bl·I_Na_ was not computed due to the short lifespan in these events.

#### 3D simulations

**Figure 9:**
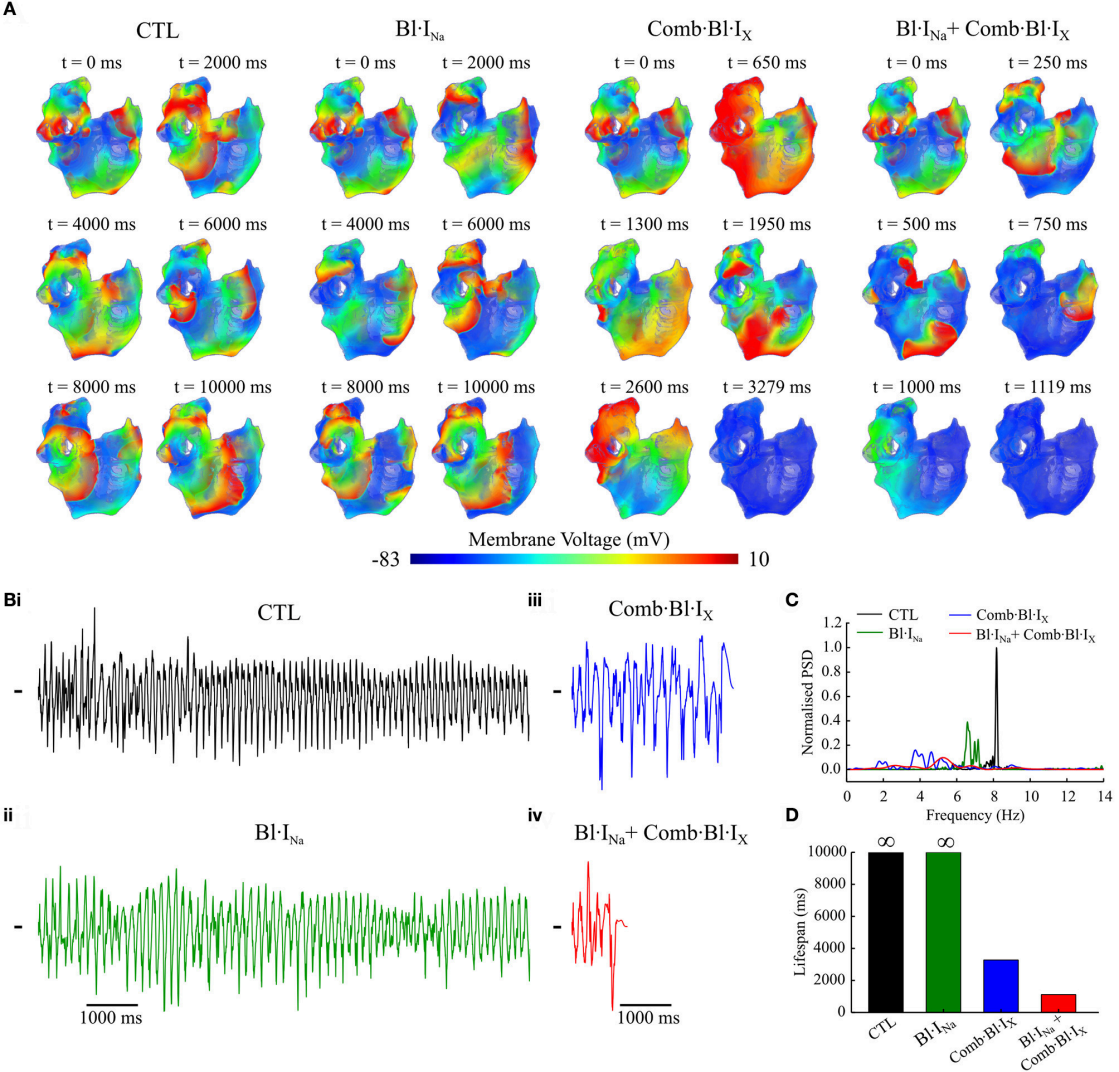
Simulated re-entrant excitations computed using the 3D anatomical model of the cAF-remodeled human atria in response to application of channel blockers as compared to that of the drug-free (CTL) condition. **(A)** Snapshots of electrical excitation waves. Drugs were applied at *t* = 0 ms. **(B)** Simulated pECGs. **(C)** Power spectrum density (PSD) obtained from the pECGs. PSDs were normalized to the maximum value of that in control. **(D)** Lifespan of re-entrant excitations in these simulated atria for various conditions. The symbol ∞ indicates the spiral waves were sustained throughout the 10-s episode of simulation. $K_{A}=1\text{ m}{\text{s}}^{-1}\cdot {\text{M}}^{-1},\;K_{I}=100\text{ m}{\text{s}}^{-1}\cdot {\text{M}}^{-1},L_{A}=1\text{m}{\text{s}}^{-1},\;L_{I}=0.01\text{ m}{\text{s}}^{-1}$;$\;\left[D_{K^{+}}\right]=3.2\mu$M, $\left[D_{Na^{+}}\right]=60\mu$M.

The antiarrhythmic effects of acacetin and Na^+^-block on the re-entrant waves in the cAF-remodeled atria were also evaluated using our 3D anatomical model of the human atria (Colman et al., 2013, 2017). A 10-s episode of sustained re-entrant excitation was first initiated in the cAF-remodeled atria in the drug-free condition; this produced the initial conditions of the 3D model for additional 10-s episode simulations. Next, the behavior of electrical waves of another 10-s episode simulation in the drug-free condition and after applying the selected blockers was analyzed and compared. Figure 9A shows snapshots of excitation wave evolution following the 10-s episode of re-entrant excitation events. pECGs computed from the excitation waves are shown in Figure 9Bi–iv. Figures 9C,D illustrates power spectrum analyses of the pECGs and a comparison of the lifespan of the electrical waves in the drug-free condition and after applying the drugs/blockers. In the drug-free condition, stable re-entrant waves around the pulmonary veins and left atrium were observed; the power spectrum density (PSD) manifests a single-focused peak around 8.16 Hz. Following applying Bl·I_Na_, the re-entrant waves became less organized and also decelerated. This was characterized by a smaller dominant frequency (6.58 Hz) and less focused PSD distribution; the excitation was not terminated. Following the application of Comb·Bl·I_X_, the re-entrant wave soon became unstable and eventually disappeared after *t* = 3,279 ms. Note that the peak PSD amplitude was much smaller and its distribution was much broader as compared to the drug-free condition. The combined drugs further destabilized the re-entrant waves and reduced the lifespan to approximately 1,120 ms.

### Simulated effects of acacetin and Na^+^-current blocker on the QT interval

**Figure 10:**
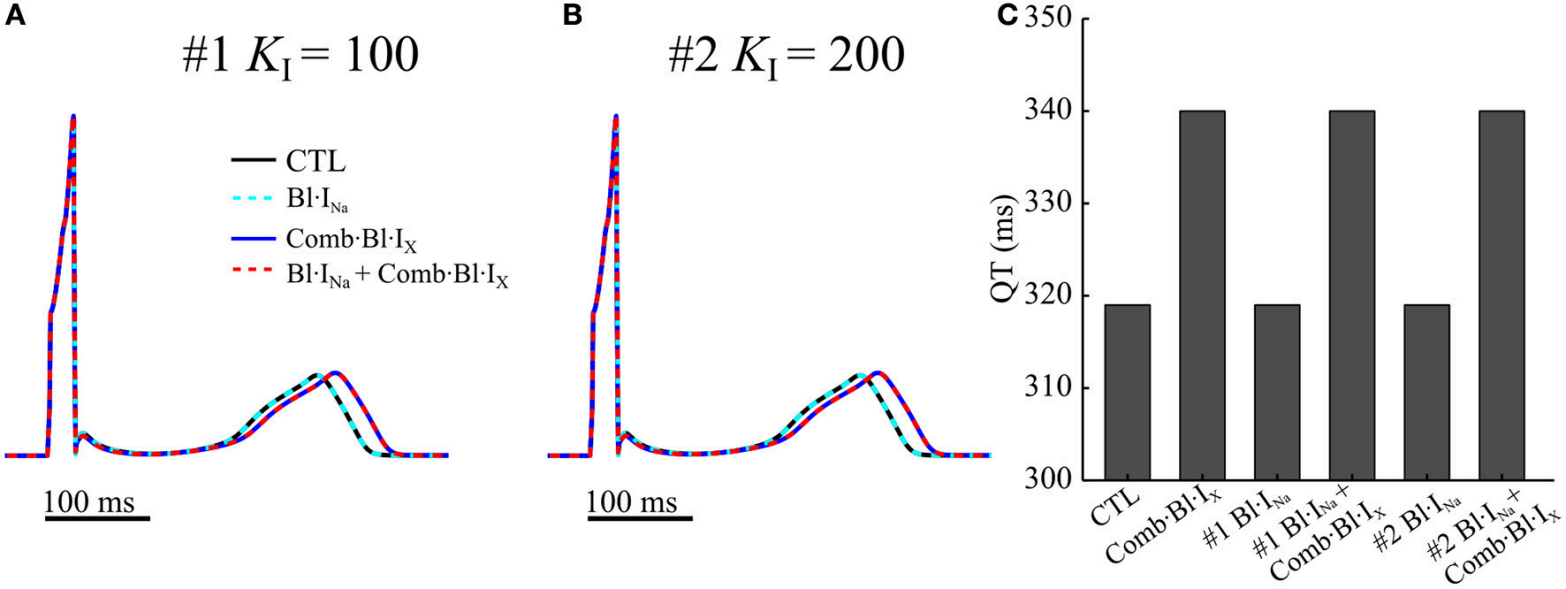
Computed pECGs from a 1D strand model for human ventricular transmural strands in drug-free (CTL) condition and in response to anti-arrhythmic drugs. In **(A,B)** two sets of parameters (#1 *K*_I_ = 100 ms^−1^·M^−1^, #2 *K*_I_ = 200 ms^−1^·M^−1^) for I_Na_ block were simulated. **(C)** QT intervals measured from the pECGs.

Further simulations were performed using a 1D ventricular transmural strand model to evaluate the changes in the waveform of electrograms in consequence of applying the drugs. A comparison of the computed electrogram waveforms are illustrated in Figures 10A,B, and the QT intervals are quantified in Figure 10C. Blocking small fractions of I_Kr_, I_Ks_ and I_to_ in the human ventricles by acacetin slightly increased the QT interval by 21 ms. The electrograms were not noticeably affected by applications of the Na^+^-blocker with the simulated parameters. Results from both cases did not show dramatic QT prolongation, indicating no dramatic effects affecting ventricular repolarization process which might promote ventricular arrhythmias.

## Discussion

Even decades after goal-directed work, successful development of effective and safe antiarrhythmic drugs for treating AF has not been accomplished and remains a major unmet clinical need. In a recent study on the canine heart, enhanced anti-arrhythmic effects and AF-selectivity of I_Na_ blockade by additional I_Kur_ block was demonstrated (Aguilar et al., 2015). Whether similar effects could be obtained in the human atria, especially following cAF-induced electrical remodeling which reduces I_Kur_, remained unclear. How the combined Na^+^ and K^+^-block modulates the QT interval also remained incompletely understood. In this study, the effects of I_Kur_ (combined with a modest block in I_to_, I_Kr_, and I_Ks_ as presented by acacetin, a compound shown to be effective in anti-AF treatment) and I_Na_ block (two potentially effective atrial-selective block on human atrial electrophysiology) were investigated *in silico* using multiscale models of the human atria and state-dependent block scheme. The simulation results demonstrate that both Na^+^-block and K^+^-block exhibited anti-arrhythmic effects in the atria following cAF-remodeling, despite reduced I_Kur_ by the remodeling. The present study highlighted that in addition to combined Na^+^- and K^+^-block, combined multi-K^+^-channels also exerted beneficial synergistic antiarrhythmic effects when compared with single channel block whilst having modest impact on ventricular repolarization (QT interval). This study suggests that multi-channel block (either combined Na^+^-K^+^-block, or combined multi-K^+^-block) may be a favorable strategy for the development of novel pharmaceutical therapies for AF.

### Effects of I_Na_ block

An atrial-ventricular difference in the properties of I_Na_, especially in the voltage dependence of steady-state inactivation, has been reported (Li et al., 2002; Burashnikov et al., 2007; Chen et al., 2016; Fan et al., 2016; Caves et al., 2017). In these studies, the voltage dependent steady-state inactivation curves for I_Na_ were found to be negatively shifted (by 5–16 mV) in atrium as compared to the ventricular parameters. This difference gave rise to an on-going interest in developing an atrial-selective blocker of I_Na_ as a strategy in terminating AF (Burashnikov et al., 2007; Antzelevitch and Burashnikov, 2009; Zygmunt et al., 2011; Morotti et al., 2016; Caves et al., 2017). As was done in previous studies (Aguilar-Shardonofsky et al., 2012; Aguilar et al., 2015), in this study, the kinetic parameters in drug actions of Na^+^-blockers were varied over wide parameter spaces to reveal AF-selectivity of Na^+^-blockers in the ventricles and fibrillating atria. Our results demonstrated that in the presence of AF-remodeling, an atrial-selective block of I_Na_ could produce different effects between atrial and ventricular cells (Figures 3, 4) and that the AF-selectivity could be maximized by optimizing the binding and unbinding rates of the Na^+^-blocker (Figure 6). Note also that, the fractional inhibition of I_Na_ by the Na^+^-blocker exhibited a substantial dependence on the rate of pacing (Figures 3, 4 and Online Supplementary Material 2.2), which was quantified using the rate-selectivity (Figure 6).

At the cellular level, Na^+^-block resulted in a significant inhibition in I_Na_ at fast pacing rates, but minor effects within the range of normal heart rates in the atria (Figures 5, 6). The antiarrhythmic effects of these changes were demonstrated in simulations of multicellular atrial tissue. In a 1D atrial strand model, applying Na^+^-block progressively enhanced the rate-adaptations of V_max_ and CV over a larger range of BCLs, whereas the atrial APD was not affected. At fast pacing rates, V_max_ and CV were decreased significantly, suggesting reduced excitabilities of atrial myocytes (Figure 5). These results are in concordance with the recent study (Aguilar et al., 2015) where similar effects of Na^+^-block on the canine atria were demonstrated *in silico* and experimentally in coronary-perfused hearts.

In 2D tissue simulations, this Na^+^-block shortened the lifespan and caused slowing in the excitation rate of the spiral waves (Figures 7, 8). In the 3D anatomical model, applying Na^+^-block alone produced antiarrhythmic effects by slowing the re-entrant excitations (Figure 9). Furthermore, in our 1D model of transmural ventricular strand, the simulations suggested that the Na^+^-block had minimal impact on ventricular repolarization, as judged by modest QT interval prolongation. These results demonstrated that Na^+^-block could be beneficial in suppressing re-entrant activities in the cAF-remodeled atria, with modest impact on ventricular repolarization.

### Effects of K^+^-current block

K^+^-current blockers delay the repolarization phase of the AP and thus prolong atrial APD and refractory period. This can cause disruptions and eventually termination of the re-entrant circuits (Hancox et al., 2016). However, K^+^-channel blockers such as dofetilide and sotalol (which potently inhibit I_Kr_) have a substantial risk of prolonging QT interval and promoting *Torsades de pointes* arrhythmias (Hondeghem and Snyders, 1990; Yap and Camm, 2003). In principle, blocking atrial-specific K^+^-channels may exert antiarrhythmic effects in the atria while minimizing potential risks of adverse effects in the ventricles. I_Kur_ is believed to be such an atrial-selective substrate for drug interventions, and effects of I_Kur_ block have been extensively studied (Burashnikov and Antzelevitch, 2008; Li et al., 2008; Tsujimae et al., 2008; Almquist et al., 2010; Pavri et al., 2012; Scholz et al., 2013; Loose et al., 2014; Ford et al., 2016). Interestingly, many existing I_Kur_ blockers potently block other K^+^-channels including I_to_ and I_K,ACh_ (Gögelein et al., 2004; Wirth et al., 2007; Burashnikov and Antzelevitch, 2008; Li et al., 2008). The additional blockades of these channels may contribute to the antiarrhythmic effects of those drugs, which warrant further investigations.

In this study, acacetin, a compound initially isolated from the traditional Chinese medicine *Xuelianhua*, was selected as a representative I_Kur_ blocker. The effects of acacetin on atrial electrophysiology were evaluated in two ways: (a) the effects of acacetin blocking I_Kur_ only; and (b) the full actions of acacetin on the targeting channels (I_to_, I_Kur_, I_Kr_, and I_Ks_) (Li et al., 2008). This approach allowed for investigations into the effects of I_Kur_ block alone as well as the potential benefits of additional-but-modest inhibition of other K^+^-currents in the human atria.

#### Selective I_Kur_ block

Blocking I_Kur_ with 3.2 μM acacetin exerted APD prolongation (9.8 ms) under the baseline/normal conditions (Figure 2). Experimental data show that dependent on the baseline AP waveform the effect of I_Kur_ block on human atrial APD_70−90_ under normal (SR) conditions can manifest as prolongation or shortening in the APD, (Workman et al., 2001; Wettwer et al., 2004; Schotten et al., 2007; Burashnikov and Antzelevitch, 2008; Loose et al., 2014). Additionally, the prolongation in APD by I_Kur_ block observed in the present study is similar to our previous paper (Colman et al., 2017) concerning the effects of genetically down-regulated I_Kur_. Moreover, inhibiting I_Kur_ under normal conditions elevated the AP plateau potential and prolonged APD_30_ (Figure 2). Both effects matched well with experimental studies (Workman et al., 2001; Wettwer et al., 2004; Schotten et al., 2007; Burashnikov and Antzelevitch, 2008; Loose et al., 2014) and our simulation study (Colman et al., 2017).

We note that in the cAF-remodeling cells, a more pronounced prolongation in APD (by 23.6 ms for 1 Hz and 16.2 ms at 6 Hz, Figure 4) was observed in the presence of 3.2 μM acacetin, despite that this current was down-regulated by cAF-remodeling (Wagoner et al., 1997; Brandt et al., 2000; Christ et al., 2008). These results are in accordance with previous experimental results of blocking I_Kur_ with MK-0448 (Pavri et al., 2012; Loose et al., 2014). In addition, I_Kur_ block exhibited enhanced rate-dependent adaptations in APD both at the cellular (Online Supplementary Material 2.2) and 1D strand models (Figure 5). Importantly, the CV restitution curve shifted toward higher BCLs, indicating that this tissue is less capable of conduction of atrial excitation waves at high rate while maintaining conduction of slow waves (Figure 5). In 2D tissue simulations, applying I_Kur_ block alone (3.2 μM acacetin) destabilized the cores of rotors (i.e., potential organizing centers for AF), and slightly slowed their excitation rates, but failed to terminate them (Figure 7), suggesting a limited efficacy of terminating AF by I_Kur_ block alone. Similarly, a recent modeling study by Aguilar et al. (2017) suggested that the antiarrhythmic efficacy of I_Kur_ block was substantially decreased in the presence of AF-induced electrical remodeling. Also, the experimental study (Burashnikov and Antzelevitch, 2008) showed that block of I_Kur_ by 4-AP of small doses had limited efficacy in suppressing AF in canine atria. This may represent the fact that I_Kur_ is reduced at high frequencies (as discussed/suggested in Feng et al., 1998a; Burashnikov and Antzelevitch, 2008; Wu et al., 2011) and shown in Figure 1) as well as by cAF-induced remodeling (Wagoner et al., 1997; Brandt et al., 2000; Christ et al., 2008). In addition, I_Kur_ is primarily active during phase 2 of AP, and hence pure I_Kur_ block exerted a relatively greater prolongation in APD_30_ than APD_90_ (Figure 3), in contrast to other K^+^-block including dofetilide which mediates anti-AF effects by prolonging the terminal phase of the AP (Roukoz and Saliba, 2007).

In this study, I_Kur_ block was simulated using a state-dependent block model, which successfully reproduced the use- and rate-dependent inhibition of acacetin (Figure 1). The rate-dependent block of I_Kur_ exerted a higher fractional inhibition in the current at faster pacing rates, which likely produces greater anti-AF effects in the presence of high-frequency excitations as seen during AF. Along with the previous modeling studies on investigating effects of I_Kur_ block (Almquist et al., 2010; Scholz et al., 2013; Ellinwood et al., 2017), this study demonstrated the importance of explicitly considering the kinetic properties of the block in computational efforts of understanding the consequences and underlying mechanisms of I_Kur_ block.

#### Effects of combined K^+^-current block

The combined K^+^-block (as exhibited by acacetin and many other I_Kur_ blockers) resulted in synergistic APD prolongation as well as an increased efficacy in terminating re-entry in tissue as compared to the pure I_Kur_ block.

Note that at the single myocyte level, the combined actions of acacetin produced greater prolongation in atrial APD than the sum of changes due to drug-induced block of individual channel in normal and cAF-remodeled myocytes (Figure 2). Additionally, the combined K^+^-block increased the rate-additivity of APD as compared to the pure I_Kur_ block (Online Supplementary Material 2.2). This was also consistently observed in the 1D simulation (Figure 5). In the setting of pure I_Kur_ block, the elevated and prolonged plateau phase of the AP could promote the activation of I_Kr_/I_Ks_, which in return may accelerate the repolarization of AP-phase 3 (Colman et al., 2017). Therefore, additional inhibition in I_Kr_ by an identical fraction is expected to result in a greater APD prolongation than a pure I_Kur_ or I_Kr_/I_Ks_ block.

In 2D simulations, the combined K^+^-block produced an enhanced efficacy in suppressing AF compared with the pure I_Kur_ block: promoting meandering of rotor tips (Figure 7B), shortening the lifespan of re-entries (Figure 8A) and slowing of spiral wave excitations (Figure 8B). Rotor meandering is one mechanism by which spiral waves may meet non-conducting boundaries to extinguish re-entry (Narayan et al., 2013; Pandit and Jalife, 2013; Rappel et al., 2015).

The effects of acacetin (3.2 μM) on the ventricular AP and QT interval was assessed in a single cell model and 1D transmural strand model by assuming similar blockade effects of the compound on the human ventricles and atria. It was shown that following applying acacetin, the ventricular repolarization and QT interval was both preserved with slight prolongations around 21 ms (Figure 10). Our results are close to the previous experimental study (Li et al., 2008) showing that QT intervals were not prolonged by acacetin in isolated rabbit hearts and anesthetised dogs.

The synergistic effects demonstrated by the combined K^+^-blocks have implications on developing novel pharmaceutical anti-AF therapies. Given that I_to_, I_Kr_ and I_Ks_ contribute to the repolarizations of ventricular APs, inhibitions in these channels may promote risks of side effects in the ventricles. In this regard, combined block of atrial-specific K^+^ channels may be favorable. Recently, another two families of K^+^-channels that are dominantly expressed in the atria have been acknowledged: the small-conductance Ca^2+^-activated K^+^ (SK) channels (I_SK_) (Qi et al., 2014), and the two-pore K^+^ (K2P3.1) channel (I_TASK−1_) (Schmidt et al., 2015), further to the well-known constitutively active acetylcholine-activated K^+^ current (I_K,ACh_). Combined block of these atrial-specific channels may exert greater and safer antiarrhythmic effects in the atria, warranting future investigations.

### Synergistic effects of combined Na^+^- and K^+^- block

The present study reveals novel and significant synergistic effects of combined block of Na^+^- and K^+^-currents (I_Na_ and pure-I_Kur_/multi-K^+^-block) and demonstrates the additional synergistic anti-arrhythmic effects derived from the multi-K^+^ channel block in cAF-remodeled atria.

In cAF-remodeled atria, combined Na^+^- and K^+^-block significantly increased the fractional I_Na_ inhibition and APD prolongation (Figures 3, 4) and promoted pronounced AP alternans at 6 Hz, with complex effects in human AF (Narayan et al., 2011). In the simulations varying the blockade kinetics of I_Na_ block, the combined block dramatically augmented the attainable maximal AF-selectivity in consequence of enhanced atrial-selectivity and rate-selectivity as compared to the pure Na^+^-block (Figure 6).

In the 1D model of an atrial strand, combined Na^+^ and K^+^-block produced synergistic reductions in V_max_ and CV; the threshold of BCL allowing a 1:1 conduction was increased as compared to the control conditions (Figure 5). In simulated re-entrant waves in 2D and 3D atria, the combined Bl·I_Na_ + Comb·Bl·I_X_ exhibited a greater efficacy in suppressing AF, with a decreased lifespan of rotors as compared to that by either individual block (Figures 7–9). Although the combined Bl·I_Na_ + Bl·I_Kur_ did not further reduce the lifespan of spiral waves as compared to the Bl·I_Na_ alone, the combination did lead to the extinction of one of the two rotors (Figures 7Aiv,Biv) and deceleration of re-entrant activations (Figure 8B). Follow-up simulations showed that consistent synergistic antiarrhythmic effects could be obtained with reduced doses of Na^+^- and K^+^- blockers.

The non-specific multi-channel blockade is increasingly recognized as a strategy for pharmaceutical therapy of AF both experimentally (Sicouri et al., 2010; Aguilar et al., 2015; Kirchhoff et al., 2015; Hartmann et al., 2016) and clinically (Koskinas et al., 2014; Reiffel et al., 2015). In a previous study (Aguilar et al., 2015), synergistic anti-arrhythmic effects were demonstrated both *in silico* and experimentally in healthy canine hearts. Additionally, the favorable synergistic antiarrhythmic effects have also been reported in combined block of I_SK_ and I_Na_ in an experimental atrial-fibrillated guinea pig model (Kirchhoff et al., 2015). Also, the recent HARMONY trial (Reiffel et al., 2015) revealed synergistic AF-suppressing effects for combined use of ranolazine and dronedarone. While revealing the synergistic effects of combined Na^+^- and K^+^- block in cAF-remodeled human atria, this study supports and adds insights into the on-going efforts in developing multi-channel block as a strategy for the treatment of AF.

### Limitations and future work

In the absence of the required detailed experimental data, when simulating effects of acacetin on I_to_, I_Ks_ and I_Kr_, the dose-dependence block of acacetin was assumed to be identical in both human atrial and ventricular cells. This assumption may warrant further investigations. In addition, the parameters of atrial I_to_ have been reported to be different from those of ventricular I_to_ in human (Amos et al., 1996). Previous studies reported that the IC_50_ of 4-AP block of atrial I_to_ was one-third of that of ventricular I_to_ (Amos et al., 1996; Nattel et al., 2000). If a similar atrial-vs.-ventricular difference in the IC_50_ of I_to_ and/or I_Kr_/I_Ks_ could exist for acacetin, the effects of acacetin on the ventricular electrophysiology would be less significant than our simulations, which might result in to a smaller change in ventricular I_Na_ and APD for the combined block of Bl·I_Na_ and Comb·Bl·I_X_, and thus enhance the computed atrial-selectivity and AF-selectivity of the combined block. Given that applying acacetin *in vivo* did not prolong QT intervals in isolated rabbit hearts and anesthetised dogs (Li et al., 2008), any significant prolongation of the ventricular APD and QT interval is unlikely (Figures 3B, 4C, 10). Therefore, our assumption of no atrial-ventricular difference in the potency of acacetin on K^+^-currents may not affect our conclusions concerning the atrial-selectivity of combined Na^+^- and K^+^-block.

Additionally, in the absence of detailed experimental data for state-dependent block of I_to_, I_Kr_, and I_Ks_ by acacetin, the block of these channels was modeled using a single pore block model. The IC50 values (Table 1) were determined by fitting the concentration-response relation of the step current at 40 mV in previous experimental studies (Li et al., 2008; Wu et al., 2011). A recent study suggests that the IC50 values may be dependent on the voltage protocols applied, and this cannot be reflected by single pore models. In future studies, the pore block model for I_to_, I_Kr_, and I_Ks_ can be replaced by a state-dependent block model when such experimental data become available. Also, the present work did not attempt to model the effects of acacetin on I_K,ACh_, although the study shows the current is potently blocked by the compound. The 2D and 3D simulations of atrial tissue, while validated, may not fully capture the complexity of fibrosis-tissue interfaces which are seen in structurally remodeled atria and were not simulated in these monodomain experiments.

Thirdly, our simulation results showed a moderate QT prolongation of around 20 ms following applying both I_Na_ blocker and acacetin. While a QT prolongation of less than 5 ms does not raise a regulatory concern (Committee for Medicinal Products for Human Use, 2012), implications of QT prolongations between 5 and 20 ms remain inconclusive (Committee for Medicinal Products for Human Use, 2005). In the present study, though the extent of QT prolongation of 20 ms is far less than the threshold of discontinuation criteria of 60 ms as indicated in Committee for Medicinal Products for Human Use (2005), it would indeed raise a positive flag in thorough QT tests and necessitate extended safety assessment and intensive patient monitoring during late stages of trials (Committee for Medicinal Products for Human Use, 2012). On the other hand, the approach we used in accounting for the effects of acacetin on ventricular myocytes may result in upper bound of QT prolongation, since the potency of acacetin was assumed to be identical in atria and ventricles.

Fourthly, the threshold in BCL inducing AP alternans was increased by K^+^-block (Figure 5, Figure S5). However, AP alternans seen at slower pacing rates has been linked with occurrence of AF (Narayan et al., 2002, 2011). Therefore, the increased threshold in BCL developing AF by K^+^-block can be potentially proarrhythmic. The safety of K^+^-block and its proarrhythmic potential in the atria should be addressed in future studies.

Fifthly, there are limitations in the approaches used in simulating the I_Na_ blockers in single myocytes and tissue. Similar to previous studies (Aguilar-Shardonofsky et al., 2012; Aguilar et al., 2015), in our simulations, the drug action on I_Na_ was modeled through a state-dependent block assuming drugs binding to both activated and inactivated states of I_Na_, and the gating variables of I_Na_ were modeled using an Hodgkin-Huxley scheme. The limitations in this approach outlined in Aguilar-Shardonofsky et al. (2012) therefore apply in the present study. The results of the use-dependent block may be affected by the models used (Aguilar-Shardonofsky et al., 2012). However, the previous study (Aguilar-Shardonofsky et al., 2012) compared this modeling scheme with simulations using a Markov model, showing qualitative agreement in major findings. Therefore, the major conclusions drawn from this study may not be affected by the selected modeling approach for I_Na_ and drug interactions. Furthermore, in optimizing the AF-selectivity of the I_Na_ and K^+^-current blockers, the concentrations of Na^+^ and K^+^ blockers were fixed at 60 uM. This may potentially impose limitations in discomposing the role of the binding parameters in the modulatory effects of the blockers because of the very slow kinetics of the drug binding to its targeted channel at this high concentration. It warrants further studies by varying the concentration of blockers to simulate the optimized effects of the AF-selectivity of I_Na_ blocker. In tissue simulations, effects of drugs were modeled by increasing their doses homogeneously, simultaneously and instantaneously. The realistic actions of I_Na_ blockers in tissue, however, may be different. Also, in tissue simulations a homogenous cell model was used. As previous study (Feng et al., 1998b) showed atria are electrically heterogeneous, future work is needed to assess how tissue heterogeneities affect the efficacy of atrial-selective pharmaceutical interventions. Furthermore, the current simulations did not take the cardiac autonomic regulation into account in order to take into considerations of acacetin on I_KACh_. Future studies on interactions of atrial-selective anti-arrhythmic drug actions and autonomic systems may also render valuable findings.

It is important to acknowledge that administration of class Ic agents for Na^+^-block can cause cardiac arrhythmia and increased mortality (Echt et al., 1991). Further investigations are therefore warranted to assess the safety of the simulated Na^+^-block in the heart, especially in the ventricles.

## Conclusions

By using state-dependent drug block models and our mathematical models of the human atria, the antiarrhythmic effects of atrial selective Na^+^- and K^+^-blockers on the cAF-remodeled atria were evaluated. The combined block of multiple K^+^-currents as well as simultaneous block of Na^+^- and K^+^-currents produced synergistic antiarrhythmic effects. Our results suggest that developing multi-channel (multiple K^+^ currents and/or combined Na^+^- and K^+^-current) block is a potentially valuable strategy for the treatment of AF.

## Author contributions

HZ and HN conceived the study. HN designed experiments, developed and validated computational models, and performed numerical experiments. HN, DW, and WW analyzed data. HN, DW, WW, WG, SN, and HZ interpreted data and wrote the manuscript.

### Conflict of interest statement

The authors declare that the research was conducted in the absence of any commercial or financial relationships that could be construed as a potential conflict of interest.
