# Supplementary material for: Synergistic Anti-arrhythmic Effects in Human Atria with Combined Use of Sodium Blockers and Acacetin
Source: Front Physiol. 2017 Nov 23;8:946. doi: 10.3389/fphys.2017.00946 (PMC5703742; doi:10.3389/fphys.2017.00946)
Supplement: Supplementary file 1 [file DataSheet1.docx]

Synergistic anti-arrhythmic effects in human atria with combined use of sodium blockers and acacetin

Haibo Ni^1,2,3^, Dominic G. Whittaker^1^, Wei Wang^1^, Wayne R. Giles^4^, Sanjiv M. Narayan^5^, Henggui Zhang^1,2,3,6^

^1^Biological Physics group, University of Manchester, Manchester, UK

^2^Space Institute of Southern China, Shenzhen, China

^3^Key Laboratory of Medical Electrophysiology, Ministry of Education, Collaborative Innovation Center for Prevention and Treatment of Cardiovascular Disease/Institute of Cardiovascular Research, Southwest Medical University, Luzhou 646000, China

^4^Faculties of Kinesiology & Medicine, University of Calgary, Calgary, Alberta, Canada

^5^Department of Medicine, Stanford University School of Medicine, Stanford, California

^6^School of Computer Science and Technology, Harbin Institute of Technology, Harbin, China

Online Supplementary Material

# Methods

## Single cell models

### Update to atrial single cell model

The Colman *et al.* model of human atrial cells [1–3] was updated and then used to simulate human atrial electrophysiology in this study. Experimental studies [4–6] showed a smaller systolic calcium level in human atrial myocytes than those simulated by Courtemanche *et al.* [7] and Colman et al. [1–3] models. In the present study we adjusted the baseline conductance of I_CaL_ to reduce the systolic calcium level. The steady-state activation of I_Kur_ in our previous study [2] was simplified based on experimental data [8]:

|  | $a_{inf}=\frac{1.0}{1.0+exp(\frac{V+5.52}{-8.81})}$ | (1) |
| --- | --- | --- |

where $a_{inf}$is the steady-state activation variable of I_Kur_, and $V$ is the membrane voltage. Accordingly the conductance of the I_Kur_ channel current (g_Kur_) was reduced by 10% as compared to the model used in [2] to counteract the small change to the activation gate of I_Kur_. The conductance of the I_Kur_ current is given by:

|  | $g_{\mathrm{Kur}}=0.00576\left( 4.51+\frac{1.90}{1.0+\exp\left( \frac{V-20.52}{-8.27} \right)} \right)$ (pA/pF). | (2) |
| --- | --- | --- |

The rest of the I_Kur_ formulation was kept identical to that described in [2]. The channel conductance G_Kur_ (i.e., not the maximal channel conductance) is presented as a product of a fixed value component (i.e., maximal channel conductance of I_Kur_) and a time-independent, voltage-dependent state variable. This followed previous studies [7,9]. The parameters of calcium handling system and the maximal amplitudes of I_NaCa_, I_NaK_, I_CaP_ and I_bNa_ presented in the original Colman *et al.* model [1] were adjusted accordingly to improve the profile of intracellular calcium transient (CaT) while preserving the morphology of the AP and homeostasis of intracellular Ca^2+^ and Na^+^. As was done in previous models [5,10], the intracellular K^+^  was fixed in the current updated model. A list of parameters of the updated model employed in this study is given in Table S1.

The simulated action potential (AP) and calcium transient (CaT) at a steady-state pacing rate of 1Hz using the updated human atrial model are shown in Figure S1, where they are compared with those produced by the original Colman *et al.* [1] and Courtemanche *et al.* [7] models. Compared with the original Colman et al. [1] model, the updated model produced a more pronounced spike-and-dome morphology, which was also demonstrated in the Courtemanche *et al.* [7] model (Figure S1 left panel). The systolic calcium level was reduced in our updated model as compared to the two previous models (Figure S1 right panel), and was closer to the values observed in [4–6]. Additionally, the relaxation phase of the CaT demonstrated in [4–6] was better reproduced by the updated model as compared to original Colman *et al.* [1] model,

| 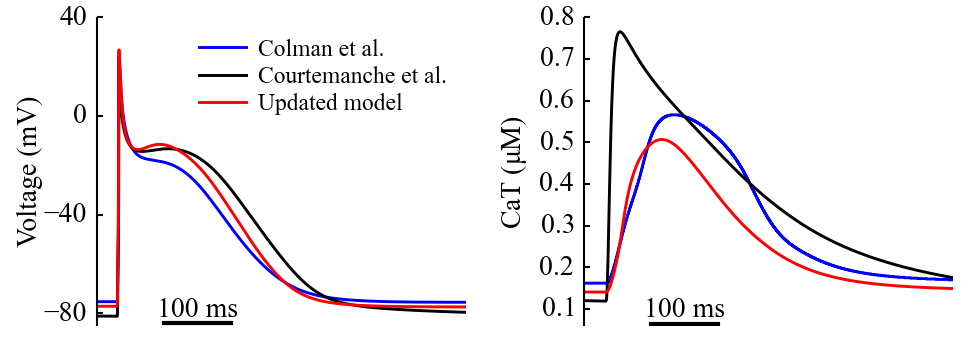  Figure S1 Simulated human atrial action potentials (AP) and calcium transients (CaT) using the updated Colman et al. model (red) as compared to those produced from the original Colman *et al.* [1] (blue) and Courtemanche *et al.* [7] (black) models. |
| --- |

### Modelling chronic-AF induced remodelling

In simulating the effects of chronic-AF (cAF) induced remodelling in the human atrial myocytes, the parameters used in our previous studies [1,2] were employed. The cAF induced remodelling in the atrial myocytes resulted in marked reductions in I_CaL_, I_Kur_ and I_to_, whereas the activity of the I_NaCa_, I_Ks_, and I_K1_ were enhanced. Under the cAF conditions, the calcium handling system was also altered; these changes included an increased RyR open probability, enhanced SR Ca^2+^ leak and reduced SERCA update rate [4]. A previous simulation study [11] demonstrated that clinically observed action potential alternans at slow pacing rates in AF [12] could be reproduced by reducing the inactivation rate of RyR. This was also incorporated into the present model by increasing the corresponding time constants of the RyR formulation. A list of parameters for the cAF model is given in Table S1.

Table S1 Parameters for the updated model of human atrial electrophysiology under SR (normal) and cAF conditions.

|  | SR conditions | cAF conditions  Relative to the SR model parameters based on [1]. |
| --- | --- | --- |
| g_bNa_, conductance of I_bNa_ | 0.000917 nS/pF | No change |
| I_Cap_, maximal I_CaP_ | 0.346 pA/pF | No change |
| I_NaCa_, maximal I_NaCa_ | 2240 pA/pF | + 40% |
| I_NaK_, maximal I_NaK_ | 1.074 pA/pF | No change |
| g_CaL,_ conductance of I_CaL_ | 0.1294 pA/pF | - 65% |
| g_to_, conductance of I_to_ | 0.1554 pA/pF | - 65% |
| g_Kur,_ conductance of I_Kur_ | Replaced with Eq. (2) | - 50% |
| g_K1_, conductance of I_K1_ | 0.09 nS/pF | + 110% |
| g_Ks_, conductance of I_Ks_ | 0.1035 nS/pF | + 100% |
| $K_{SR,leak}$, SR leak rate | 3.0e-3 ms^-1^ | + 50% [4] |
| $D_{Ca}$, Ca^2+^ diffusion between the subspace and cytosol. | 1170 μm/s | No change |
| SERCA uptake rate | Reduced by 25% as compared to [1,2] | - 40% [4] |
| RyR release | Identical to [1,2] | + 200% |
| $\tau_{RyRadapt}$ | 250 ms | + 170% |
| $\tau_{RyRact,cytosol}$ | 5 ms | No change |
| $\tau_{RyRact,subspace}$ | 5 ms | No change |
| $\tau_{RyRinact,cytosol}$ | 30 ms | + 170% |
| $\tau_{RyRinact,subspace}$ | 15 ms | + 170% |

### Rate constants of I_Kur_ block by acacetin

The binding and unbinding parameters for the interaction between drugs and ion channels were obtained by fitting the model to the experimental data on the rate-dependent blockade of I_Kur_ by acacetin [13]. When fitting the parameters, a cost function was defined as the difference between the simulated and experimental relative-current against the pulse number. The fitted parameters were obtained by minimising the cost function using the parameter optimisation procedure described in [14]. These parameters are listed in Table S2.

### Modelling human ventricular electrophysiology

The O’Hara *et al.* [15] mathematical model of human ventricular electrophysiology was employed to simulate the effects of anti-AF drugs on the ventricular AP, I_Na_, and QT interval in tissue. A recent comparative study on the behaviours of human ventricular models in tissue [16] demonstrated that the baseline O’Hara *et al.* model produced a much smaller upstroke velocity of AP than the experimental data, suggesting a limitation in the I_Na_ formulation used in the model. Therefore, in the present study the I_Na_ formulation in the model was replaced with that employed by Luo and Rudy [17]. The single ventricular myocyte simulations were performed using the endocardium myocyte model of the O’Hara *et al.* model.

Table S2 Fitted parameters describing the binding and unbinding kinetics of acacetin on the K_V_1.5 channel.

| Parameter | Value | Unit |
| --- | --- | --- |
| *K*_O_ | 0.000194 | μM^-1^⋅ ms^-1^ |
| *L*_O_ | 0.000291 | ms^-1^ |
| *Z*_KO_ | 0.257 |  |
| *Z*_LO_ | -0.0128 |  |
| *K*_C_ | 0.00249 | μM^-1^⋅ ms^-1^ |
| *L*_C_ | 0.000286 | ms^-1^ |
| *Z*_KC_ | 0.327 |  |
| *Z*_LC_ | 0.837 |  |

## Tissue models

Propagation of the excitation wave in these human tissues was described using the well-known monodomain equation [18]. The monodomain equation is given by

|  | $\frac{\partial V_{m}}{\partial t}=\nabla\cdot\left( \mathbf{D}\nabla V_{m} \right)-\frac{I_{\mathrm{ion}}}{C_{m}}$ | (3) |
| --- | --- | --- |

where ***D*** is the tensor of diffusion coefficient, *I*_ion_ the total ionic transmembrane current in a single cell and the $C_{m}$ is the membrane capacitance of the cell. In this study, tissue models ranging from 1D models representing myocardial fibres to 3D models with realistic anatomical geometries of the atria were used.

### One-dimensional models

Simulations on 1D tissue strand were performed to evaluate the effects of the drugs on the atria and ventricles. The atrial 1D strand consisted of 100 nodes of same cell type, while the 1D ventricular-strand tissue model was composed of 100 nodes that were segmented into endocardium, M-cells and epicardium cells with a ratio of 25:35:40, which was an identical implementation to a previous study [19]. In both strand models, the nodes were spaced 0.15 mm apart.

The diffusion coefficient tensor in the strand models was selected so that the conduction velocity (CV) of the simulated excitation wave propagation was close to previous studies and published experimentally determined values. In the atrial strand model, the simulated CV under normal conditions was 0.70 m/s based on our previous studies [1,2,20]. The simulated ventricular CV under normal conditions was 0.60 m/s, which was close to the previous study [19].

### Two-dimensional models

2D simulations were performed on a 7.5×7.5 cm^2^ slab representation of human atrial myocytes with 250 nodes along each direction. Re-entrant excitations were initiated within the 2D tissue to facilitate studies on the antiarrhythmic effects of the channel blockades. The diffusion coefficient ***D*** was chosen so that the model gave a conduction velocity of 0.7 m/s for plane wave in the tissue under the normal conditions. In simulations of atrial tissue under AF conditions, cAF induced remodelling to the electrical coupling was incorporated by reducing the diffusion tensor coefficient ***D*** to 80% of that under normal conditions, accounting for the cAF-induced remodelling in electrical coupling and atrial structure [1,21].

### Three-dimensional models

Our previously developed 3D human atrial model [1,2,20] (Figure S2) was applied to simulate the re-entrant excitation dynamics in tissue with realistic anatomical structure under various conditions. Detailed descriptions of the 3D model have been given in the previous studies [1,2,20]. Briefly, the human atrial geometry was segmented from the visible human dataset [22] and labelled based on the major differentiated anatomical regions [22]. Anisotropic myofibre structure in the major regions of the atria was taken into account by incorporating rule-based fibres along the bundles of the crista terminalis (CT), pectinate muscles (PM) and Buchman’s Bundle (BB), as implemented in previous studies [1,20,23]. An anisotropic ratio (9:1) in directions longitudinal and transverse to the fibre bundles was applied in these regions, which corresponded to conduction velocities of 1.3 ms^-1^ measured along fibres and 0.7 ms^-1^ transverse to the fibres under the normal conditions. In simulating cAF conditions, the diffusion tensor coefficient ***D*** was reduced by 20% as compared to that under normal conditions, accounting for a reduced electrical coupling in tissue by the cAF remodelling [1,21].

| **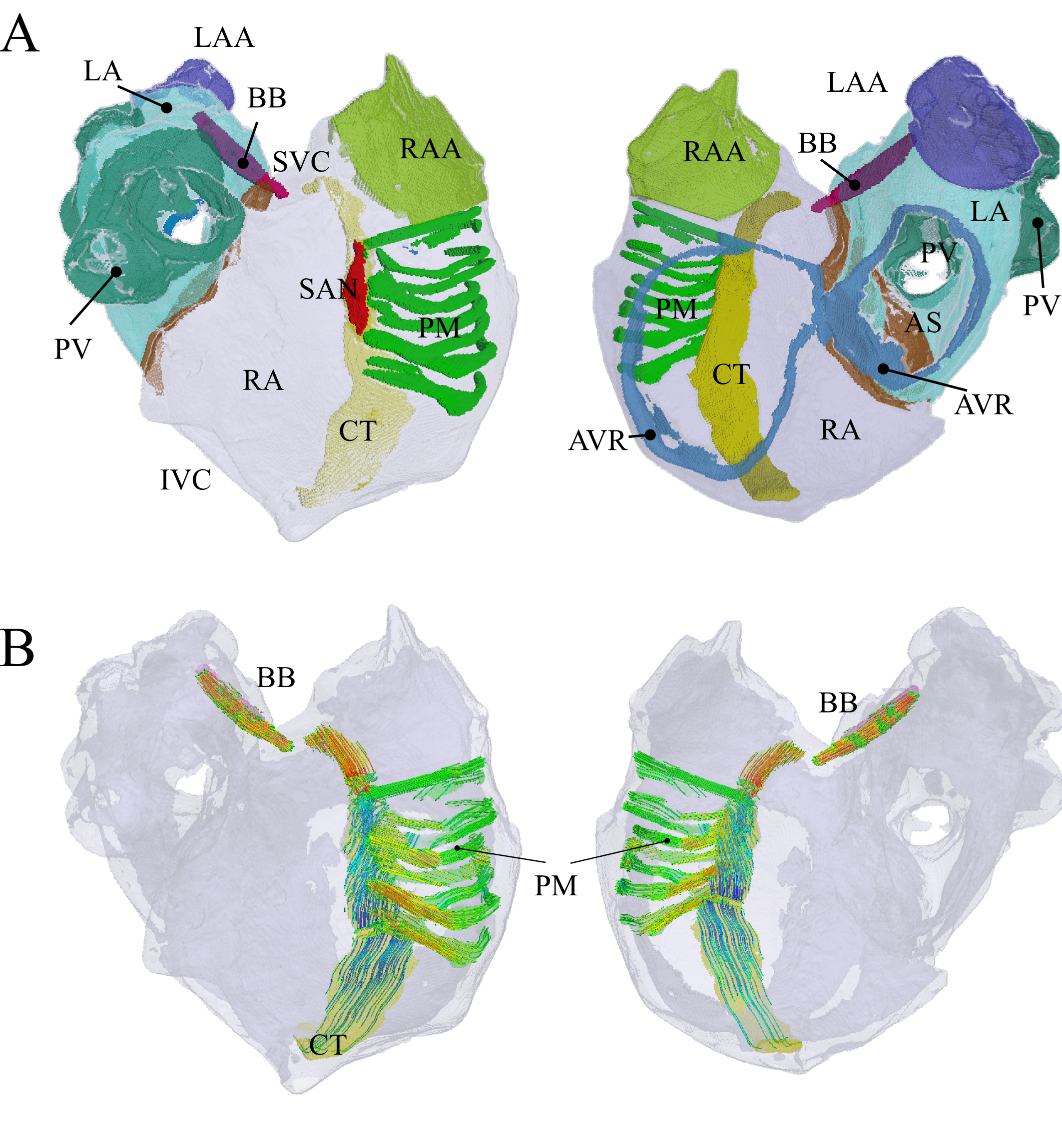**  Figure S2 Structural features of the 3D atrial model.  (A) Anatomical reconstruction of the human atria; the major functionally distinct regions are labelled. (B) Fibre orientations in the CT, PM and BB regions. CT - crista terminalis, BB - Bachmann’s bundle, PM - pectinate muscles, AVR - atrio-ventricular ring, RA – right atrium, RAA - right atrial appendage, AS - atrial septum, LA - left atrium, LAA - left atrial appendage, PV -pulmonary veins. |
| --- |

### Modelling re-entrant excitation in tissue

To initiate re-entrant waves in 2D slab tissue, the S1-S2 cross-shock protocol [24] was used: a train of S1-stimuli were first applied to the left lateral of the 2D slab tissue to allow for propagation of a planar wave (Figure S3A); a further stimuli of the same strength and duration as the S1 was applied to the bottom half of the tissue with a temporal delay of duration S2, representing a cross-shock (Figure S3B). A snapshot of an initiated re-entry by implementing the protocol is shown in Figure S3C.

| 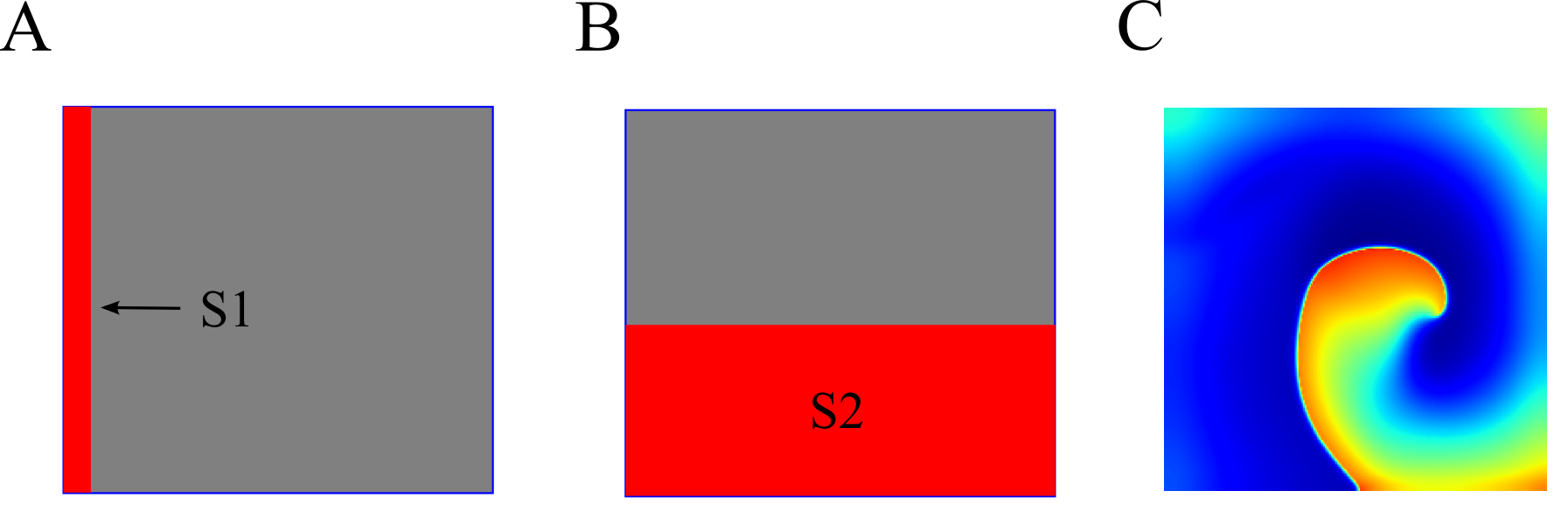  Figure S3 An illustration of the cross-shock protocol for initiating spiral waves in the atria. (A) A train of S1 stimuli were applied to one end of the 2D tissue. (B) After a delay of S2, a further stimulus was applied to the bottom-half of the 2D tissue. (C) A screen shot showing a simulated re-entrant wave initiated using the cross-shock protocol. |
| --- |

To reach steady-state conditions in the tissue model, the state variables of the myocytes in the tissue model were pre-conditioned with steady-state values from single cell modelling: an *in silico* cell was paced at 6 Hz to produce steady-state variables, which were loaded into the tissue model as initial conditions.

In 3D models, re-entrant excitations were initiated using a phase distribution method [2,25,26]. The phase distribution method applies initial conditions to the state variables of each node of the tissue model in a location-dependent manner: 1) a phase map resembling AP distribution during re-entrant wave excitation in tissue was first applied to each node of the tissue model; 2) a complete cycle of the AP paced at 6 Hz was divided into 205 segments, producing 205 phase-indexed files containing the state variables of various stages during the AP; 3) these state variable files were mapped onto the tissue model based on the predefined phase map. A detailed description of the method can be found in [2]. The phase distribution method allows for the investigation of the long term dynamics of re-entrant excitation independent of how the re-entrant waves were initiated [2].

## Analysis of re-entrant excitation events

Dominant frequency was calculated as a measure of the speed of the re-entrant excitation wave in tissue. To quantify the dominant frequency of the simulated rotors in tissue, pseudo-ECGs (pECGs) were computed using the equation as follows [27,28]

|  | $\phi\left( x^{'},y^{'}, z^{'} \right)=D\int[-\nabla V_{m}\cdot\left( \nabla\frac{1}{r} \right)]dΩ$ | (4) |
| --- | --- | --- |
|  | $r=\sqrt{\left( x-x^{'} \right)^{2}+\left( y-y^{'} \right)^{2}+\left( z-z^{'} \right)^{2}}$ | (5) |

where $\nabla V_{m}$ is the spatial gradient of the transmembrane potential and r is the distance measured from a point  source $\left( x,y, z \right)$to the coordinate of a virtual electrode $\left( x^{'},y^{'}, z^{'} \right)$; $Ω$ indicates integrations throughout the domain of the tissue. In this study, $\phi$ was computed from a virtual electrode located 25 mm to the right of the tissue slab models or close to the centre of the right atrium in the 3D simulations. pECG traces were analysed using the Fast Fourier Transform (FFT) to obtain the dominant frequencies of the simulated re-entrant excitations.

Additionally, the phase singularity detection method proposed by Bary and Wikswo [29] was adopted to trace the tip trajectories of the rotors in 2D simulations. The tip trajectories analysis enables a better understanding of the stability of self-perpetuating re-entrant excitations in tissue.

## Numerical Methods

The single cell models were solved using a combination of the Forward Euler method and the Rush-Larsen scheme [30], as implemented in previous studies [2,7]. In modelling effects of drugs, the in-silico cells were paced for further 60 s following a simulated application of drugs after a steady-state had been reached. The tissue models were discretised into structured geometry grid; the monodomain equation (Eq. (3)) was solved using a finite-difference PDE solver based on the explicit Forward Euler scheme and Strang splitting scheme [31].

# Supplemental Results

## Simulated atrial electrophysiological properties in normal and cAF-remodelled atrial myocytes

The updated model of the human atrial myocyte was first used to simulate APs and calcium transients (CaTs) in both normal and cAF-remodelled myocytes (Figure S4). The AP obtained from the updated model shows a pronounced spike-and-dome morphology.

The incorporation of cAF-induced remodelling properties to the baseline model significantly abbreviated APD (Figure S4A, 247 ms in normal vs. 157 ms in AF-remodelled myocytes), and hyperpolarised the resting potential, whilst the AP exhibited a typical triangular morphology. The amplitude of CaT was also markedly reduced with cAF-remodelling (Figure S4B). These results are well aligned with previous experimental [4,32,33] and modelling [1,5,34,35] studies. The APD restitution attained by steady-state pacing is shown in Figure S4C. The updated model under the normal conditions showed increased rate-adaptation of the APD in comparison to the original Colman et al. model [1]. The inclusion of cAF-remodelling effects significantly flattened the APD restitution and decreased APD across the entire range of simulated BCLs, which is in good agreement with previous studies [32,36,37]. At BCLs of 150 to 200 ms, alternans in AP were observed in the simulations of cAF-remodelled myocytes (Figure S4C).

| **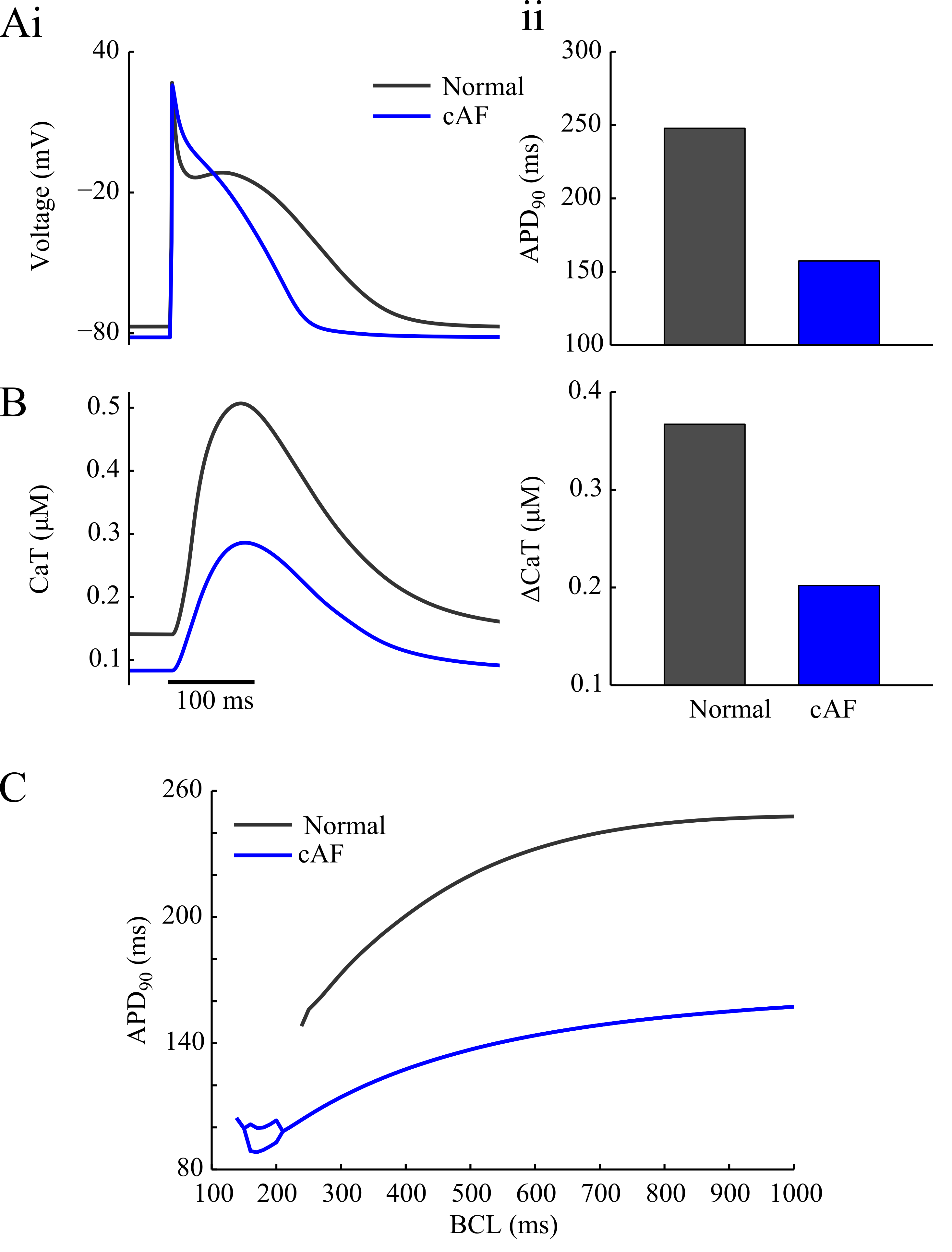**  Figure S4 Simulated action potentials (AP) and calcium transient (CaT) of normal or cAF-remodelled human atrial cardiomyocytes. (A-B) The action potential duration measured at 90% repolarization (APD_90_) and the amplitude of CaT are quantified and shown in the right panels. The cells were paced at 1 Hz. (C) Simulated steady-state APD restitution curve of normal and cAF-remodelled atrial myocytes. |
| --- |

## Simulated effects of channel block on atrial restitutions in cAF-remodelled myocytes

Figure S5 shows the effects of different combinations of Na^+^- and K^+^- block on the APD restitution properties and rate-dependence of I_Na_ in cAF-remodelled atrial myocytes. As expected, APD prolongation was observed consistently over the entire range of simulated BCLs for Bl⋅I_Kur_ and Comb⋅Bl⋅I_X_ as compared to the control conditions; the rate-dependent adaptation of APD was also promoted. Whilst Bl⋅I_Na_ alone had little effect for slow pacing rates, it induced a slight prolongation at short BCLs (Figure S5A). The reduction in peak I_Na_ in Bl⋅I_Na_ was rate-dependent and significantly greater at fast pacing rates. K^+^- block alone (Bl⋅I_Kur_ or Comb⋅Bl⋅I_X_) slightly shifted the rate-dependence of peak I_Na_ to larger BCLs via prolonging the APD without affecting peak I_Na_ at slow pacing rates. Synergistic reductions in peak I_Na_ at small BCLs was observed for combined blocks of Bl⋅I_Na_ with Bl⋅I_Kur_ or Comb⋅Bl⋅I_X_ as compared to single channel blocks. AP alternans were observed in control and became more pronounced with K^+^-current block, and further enhanced by combined Bl⋅I_Na_ and K^+^-blocks. In addition, the threshold of BCL allowing alternans was increased in these conditions as compared to control (Figure S5B).

| **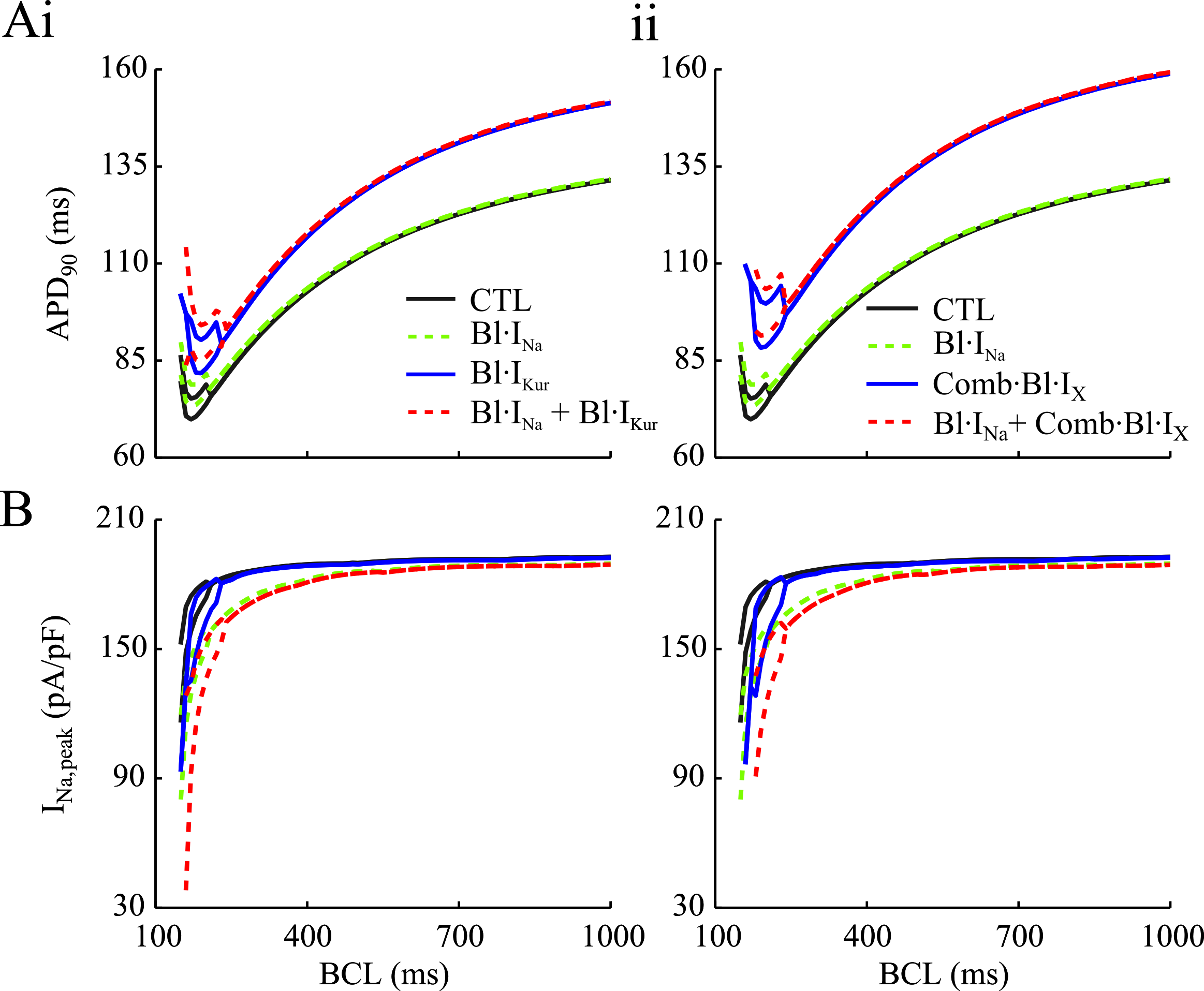**  Figure S5 Single myocyte APD restitution curves and rate-adaptations of peak I_Na_ in the drug-free (CTL) condition and after applying the selected individual or combined blockers. (A) Steady-state APD restitution curves. (B) Rate-adaptations of peak I_Na_ elicited during APs. For the ease of comparison with control and Bl⋅I_Na_, results of Bl⋅I_Kur_ and Comb⋅Bl⋅I_X_ are plotted on the left and right panels, respectively. |
| --- |

## Dependence of AF-selectivity of I_Na_ blockers on L_A_ and L_I_

To assess the dependence of the block efficacy, rate-selectivity, atrial-selectivity and AF-selectivity of I_Na_ block on the drug action kinetics, the unbinding constants $L_{A}$ and $L_{I}$were first varied over a parameter space from ${10}^{-5}$ to ${10}^{0}$ ms^-1^ while $K_{A}$ and $K_{I}$were both fixed. The results are shown in Figure S6. As expected, the block efficacy decreased with increase of L_I_ and L_A_, but to a much smaller extent with the latter. The rate-selectivity and atrial-selectivity in general exhibited higher dependence on L_I_, with the selectivity of those drugs with L_I_ = 0.01 ms^-1^ exerted higher values in these measures. Collectively, the AF-selectivity peaks at {L_A_, L_I_} = {1, 0.01} ms^-1^. These results demonstrate that AF-selectivity can be optimised by varying the parameters of drug actions.

**
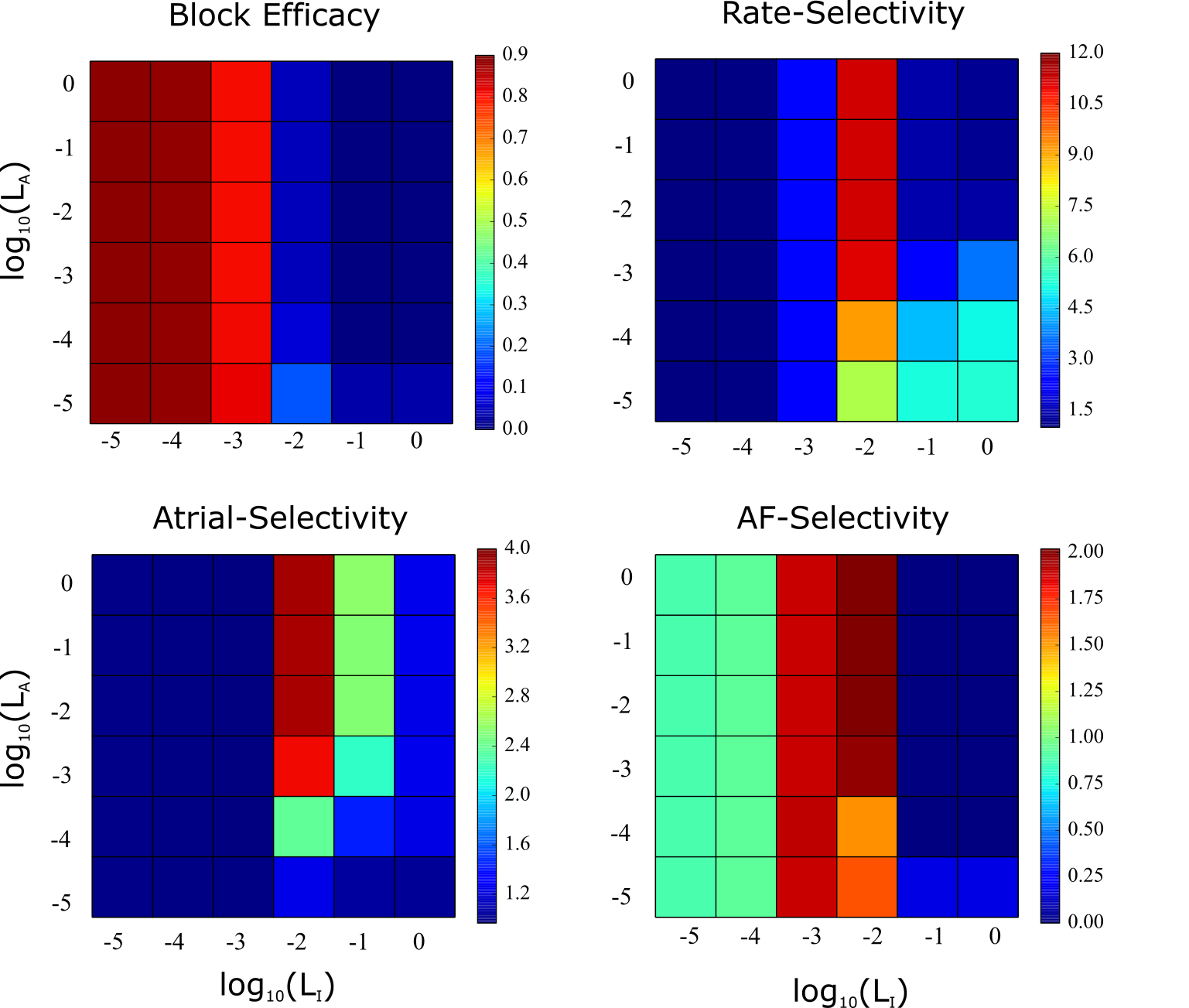
**

Figure S6 Block efficacy, and the rate-, atrial- and AF-selectivity as a function of the open- and inactivated- state binding rates ($L_{A},L_{I}$) for individual or combined Na^+^- and K^+^-block computed from cAF-remodelled atrial myocytes or ventricular myocytes.$K_{A}= K_{I}=100 ms^{-1}\cdot M^{-1}$.

## Time courses of AP, pseudo-ECG and fractional block in 2D simulations using models of cAF-remodelled atrial tissue

The electrical activity of a selected myocyte close to the bottom left of the slab was recorded and analysed. Figure S7 and Figure S8 illustrate the simulated pECGs, membrane voltage traces extracted from a local cell and the corresponding total fractional block (sum of open- and inactivated-/closed- state blocks) of I_Na_ and I_Kur_ measured from the same myocyte under selected conditions. Consistent with the results in the re-entrant wave dynamics, the AP and pECG in the baseline simulation exhibited an alternating but relatively organised pattern, whereas the corresponding measures observed following (i) I_Na_ block alone or (ii) combined channel block progressively became irregular and diminished within the simulated 10-s episode. In the simulations incorporating Na^+^-block, the total fraction of blocked I_Na_ transiently increased to up to 33% and was not affected by the further introduction of K^+^-blocks. In simulations, with administration of K^+^-blocks, the maximal total fractional block in I_Kur_ was 72% for Comb⋅Bl⋅I_X_ or Bl⋅I_Kur_ and was slightly smaller with combined actions of Bl⋅I_Na_ (69% for Bl⋅I_Kur_ + Bl⋅I_Na_ and 68% for Comb⋅Bl⋅I_X_ + Bl⋅I_Na_). Note these fractional blocks in I_Kur_ were substantially greater than 50% suggested by the dose-inhibition relation around 3.2 μM, which was attributed to the characteristics of rate-dependent block of I_Kur_ by acacetin.

| **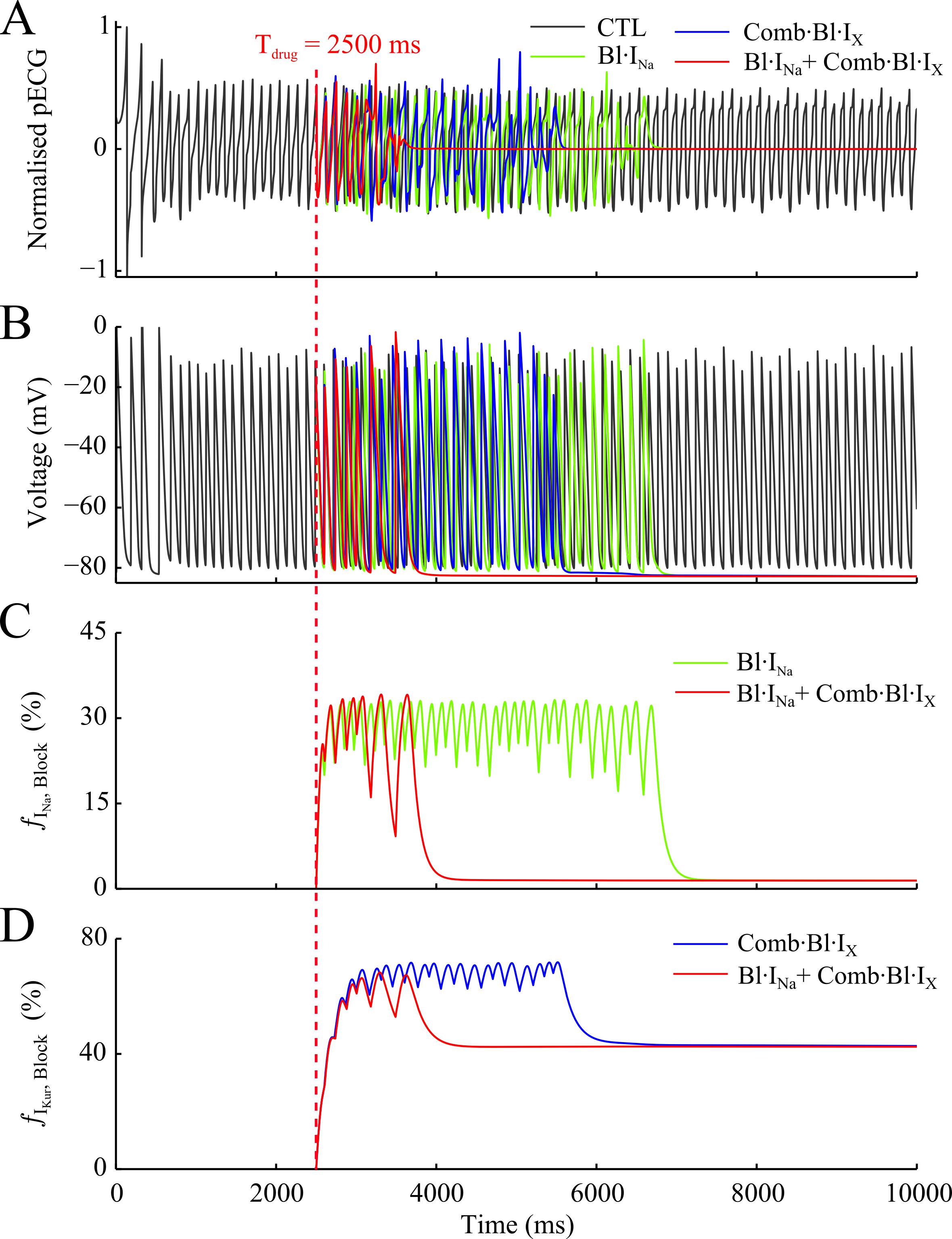**  Figure S7 Computed pECGs, time courses of membrane potential and total fractional block of I_Na_ and I_Kur_ extracted from a selected representative myocyte in drug-free (CTL) or following individual/combined block of K^+^-currents by acacetin and I_Na_ block. (A) pECGs. (B) Membrane voltage. (C) Fractional block of I_Na_ computed from the sum of open- and inactivation-state blocks. (D) Fractional block of I_Kur_ computed from the sum of open- and close-state blockade. |
| --- |
| 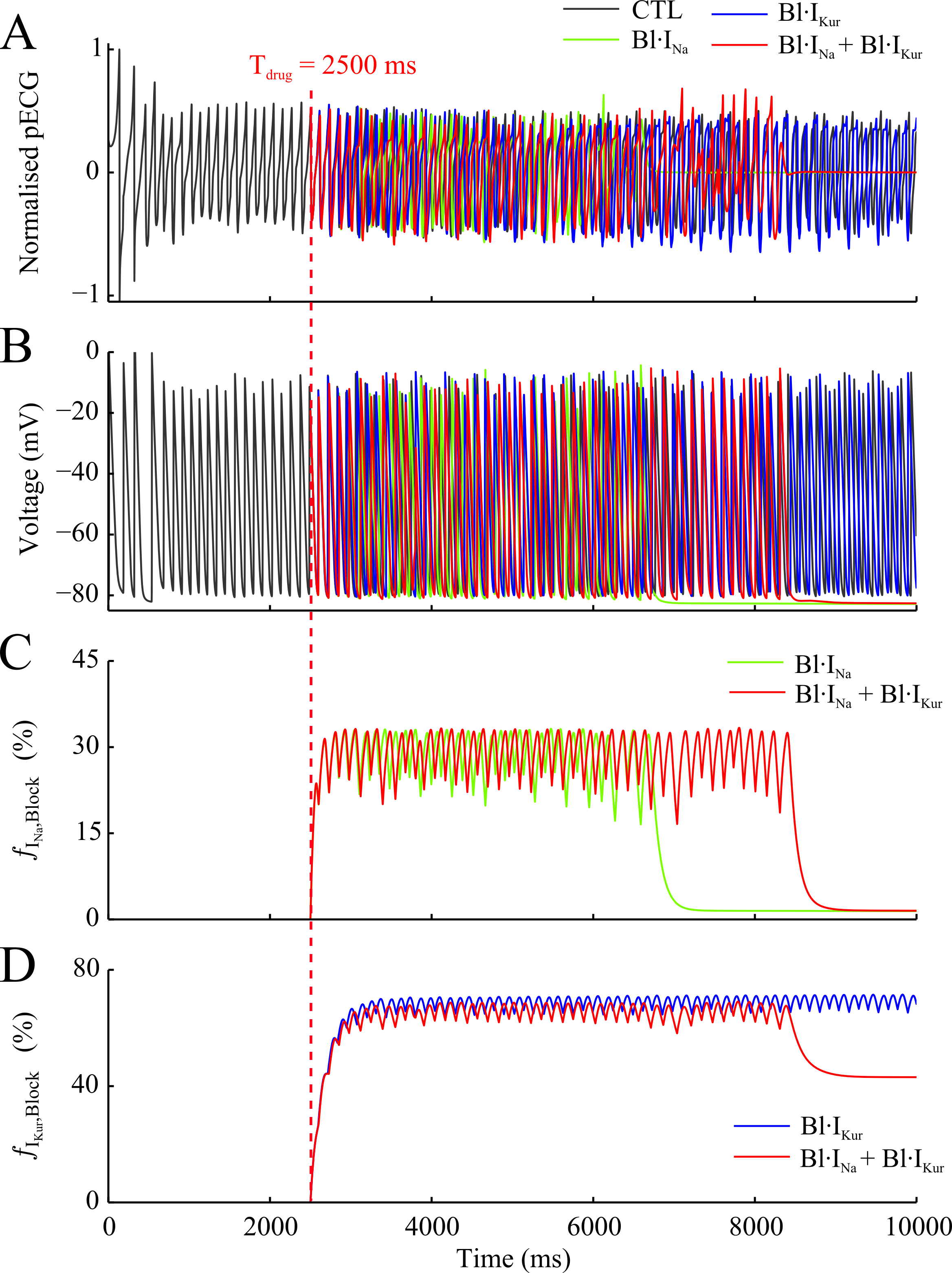  Figure S8 Computed pECGs, time courses of membrane potential and total fractional block of I_Na_ and I_Kur_ extracted from a selected representative myocyte in drug-free (CTL) or following individual/combined block of I_Kur_ by acacetin and I_Na_ block. (C) Fractional block of I_Na_ computed from the sum of open- and inactivation-state blocks. (D) Fractional block of I_Kur_ computed from the sum of open- and close-state blocks. |

# References

[1] M.A. Colman, O.V. Aslanidi, S. Kharche, M.R. Boyett, C. Garratt, J.C. Hancox, H. Zhang, Pro-arrhythmogenic effects of atrial fibrillation-induced electrical remodelling: insights from the three-dimensional virtual human atria, J. Physiol. 591 (2013) 4249–4272. doi:10.1113/jphysiol.2013.254987.

[2] M.A. Colman, H. Ni, B. Liang, N. Schmitt, H. Zhang, In silico assessment of genetic variation in KCNA5 reveals multiple mechanisms of human atrial arrhythmogenesis, PLOS Comput. Biol. 13 (2017) e1005587. doi:10.1371/journal.pcbi.1005587.

[3] D.G. Whittaker, H. Ni, A.E. Harchi, J.C. Hancox, H. Zhang, Atrial arrhythmogenicity of KCNJ2 mutations in short QT syndrome: Insights from virtual human atria, PLOS Comput. Biol. 13 (2017) e1005593. doi:10.1371/journal.pcbi.1005593.

[4] N. Voigt, N. Li, Q. Wang, W. Wang, A.W. Trafford, I. Abu-Taha, Q. Sun, T. Wieland, U. Ravens, S. Nattel, X.H.T. Wehrens, D. Dobrev, Enhanced sarcoplasmic reticulum Ca2+ leak and increased Na+-Ca2+ exchanger function underlie delayed afterdepolarizations in patients with chronic atrial fibrillation, Circulation. 125 (2012) 2059–2070. doi:10.1161/CIRCULATIONAHA.111.067306.

[5] E. Grandi, S.V. Pandit, N. Voigt, A.J. Workman, D. Dobrev, J. Jalife, D.M. Bers, Human atrial action potential and Ca2+ model sinus rhythm and chronic atrial fibrillation, Circ. Res. 109 (2011) 1055–1066. doi:10.1161/CIRCRESAHA.111.253955.

[6] N. Voigt, J. Heijman, Q. Wang, D.Y. Chiang, N. Li, M. Karck, X.H.T. Wehrens, S. Nattel, D. Dobrev, Cellular and molecular mechanisms of atrial arrhythmogenesis in patients with paroxysmal atrial fibrillation, Circulation. 129 (2014) 145–156. doi:10.1161/CIRCULATIONAHA.113.006641.

[7] M. Courtemanche, R.J. Ramirez, S. Nattel, Ionic mechanisms underlying human atrial action potential properties: insights from a mathematical model, Am. J. Physiol. - Heart Circ. Physiol. 275 (1998) H301–H321.

[8] G.-R. Li, J. Feng, L. Yue, M. Carrier, S. Nattel, Evidence for Two Components of Delayed Rectifier K+ Current in Human Ventricular Myocytes, Circ. Res. 78 (1996) 689–696. doi:10.1161/01.RES.78.4.689.

[9] M. Aguilar, J. Feng, E. Vigmond, P. Comtois, S. Nattel, Rate-dependent role of IKur in human atrial repolarization and atrial fibrillation maintenance, Biophys. J. 112 (2017) 1997–2010. doi:10.1016/j.bpj.2017.03.022.

[10] E. Grandi, F.S. Pasqualini, D.M. Bers, A novel computational model of the human ventricular action potential and Ca transient, J. Mol. Cell. Cardiol. 48 (2010) 112–121. doi:10.1016/j.yjmcc.2009.09.019.

[11] K.C. Chang, J.D. Bayer, N.A. Trayanova, Disrupted calcium release as a mechanism for atrial alternans associated with human atrial fibrillation, PLOS Comput Biol. 10 (2014) e1004011. doi:10.1371/journal.pcbi.1004011.

[12] S.M. Narayan, M.R. Franz, P. Clopton, E.J. Pruvot, D.E. Krummen, Repolarization alternans reveals vulnerability to human atrial fibrillation, Circulation. 123 (2011) 2922–2930. doi:10.1161/CIRCULATIONAHA.110.977827.

[13] H.-J. Wu, W. Wu, H.-Y. Sun, G.-W. Qin, H.-B. Wang, P. Wang, H.K. Yalamanchili, J. Wang, H.-F. Tse, C.-P. Lau, P.M. Vanhoutte, G.-R. Li, Acacetin causes a frequency- and use-dependent blockade of hKv1.5 channels by binding to the S6 domain, J. Mol. Cell. Cardiol. 51 (2011) 966–973. doi:10.1016/j.yjmcc.2011.08.022.

[14] J.D. Moreno, T.J. Lewis, C.E. Clancy, Parameterization for in-silico modeling of ion channel interactions with drugs, PLOS ONE. 11 (2016) e0150761. doi:10.1371/journal.pone.0150761.

[15] T. O’Hara, L. Virág, A. Varró, Y. Rudy, Simulation of the undiseased human cardiac ventricular action potential: model formulation and experimental validation, PLoS Comput Biol. 7 (2011) e1002061. doi:10.1371/journal.pcbi.1002061.

[16] M.M. Elshrif, E.M. Cherry, A quantitative comparison of the behavior of human ventricular cardiac electrophysiology models in tissue, PLOS ONE. 9 (2014) e84401. doi:10.1371/journal.pone.0084401.

[17] C.H. Luo, Y. Rudy, A dynamic model of the cardiac ventricular action potential. I. Simulations of ionic currents and concentration changes., Circ. Res. 74 (1994) 1071–1096. doi:10.1161/01.RES.74.6.1071.

[18] R.H. Clayton, O. Bernus, E.M. Cherry, H. Dierckx, F.H. Fenton, L. Mirabella, A.V. Panfilov, F.B. Sachse, G. Seemann, H. Zhang, Models of cardiac tissue electrophysiology: Progress, challenges and open questions, Prog. Biophys. Mol. Biol. 104 (2011) 22–48. doi:10.1016/j.pbiomolbio.2010.05.008.

[19] I. Adeniran, M.J. McPate, H.J. Witchel, J.C. Hancox, H. Zhang, Increased vulnerability of human ventricle to re-entrant excitation in herg-linked variant 1 short qt syndrome, PLoS Comput Biol. 7 (2011) e1002313. doi:10.1371/journal.pcbi.1002313.

[20] O.V. Aslanidi, M.A. Colman, J. Stott, H. Dobrzynski, M.R. Boyett, A.V. Holden, H. Zhang, 3D virtual human atria: A computational platform for studying clinical atrial fibrillation, Prog. Biophys. Mol. Biol. 107 (2011) 156–168. doi:10.1016/j.pbiomolbio.2011.06.011.

[21] S.P.J. Krul, W.R. Berger, N.W. Smit, S.C.M. van Amersfoorth, A.H.G. Driessen, W.J. van Boven, J.W.T. Fiolet, A.C.G. van Ginneken, A.C. van der Wal, J.M.T. de Bakker, R. Coronel, J.R. de Groot, Atrial Fibrosis and Conduction Slowing in the Left Atrial Appendage of Patients Undergoing Thoracoscopic Surgical Pulmonary Vein Isolation for Atrial Fibrillation, Circ. Arrhythm. Electrophysiol. 8 (2015) 288–295. doi:10.1161/CIRCEP.114.001752.

[22] G. Seemann, C. Höper, F.B. Sachse, O. Dössel, A.V. Holden, H. Zhang, Heterogeneous three-dimensional anatomical and electrophysiological model of human atria, Philos. Transact. A Math. Phys. Eng. Sci. 364 (2006) 1465–1481. doi:10.1098/rsta.2006.1781.

[23] G. Seemann, R. Moss, A.K.E. Kurz, O. Dossel, M. Tristani-Firouzi, F.B. Sachse, A 2-state Markov model of IKACh based on a membrane voltage dependent muscarinic M2 receptor approach, in: Comput. Cardiol. CinC 2012, 2012: pp. 241–244.

[24] M. Aguilar, F. Xiong, X.Y. Qi, P. Comtois, S. Nattel, Potassium channel blockade enhances atrial fibrillation-selective antiarrhythmic effects of optimized state-dependent sodium channel blockade, Circulation. 132 (2015) 2203–2211. doi:10.1161/CIRCULATIONAHA.115.018016.

[25] V.N. Biktashev, A.V. Holden, Reentrant waves and their elimination in a model of mammalian ventricular tissue, Chaos Woodbury N. 8 (1998) 48–56. doi:10.1063/1.166307.

[26] S.R. Kharche, T. Stary, M.A. Colman, I.V. Biktasheva, A.J. Workman, A.C. Rankin, A.V. Holden, H. Zhang, Effects of human atrial ionic remodelling by β-blocker therapy on mechanisms of AF: a computer simulation, Eur. Eur. Pacing Arrhythm. Card. Electrophysiol. J. Work. Groups Card. Pacing Arrhythm. Card. Cell. Electrophysiol. Eur. Soc. Cardiol. 16 (2014) 1524–1533. doi:10.1093/europace/euu084.

[27] K. Gima, Y. Rudy, Ionic current basis of electrocardiographic waveforms a model study, Circ. Res. 90 (2002) 889–896. doi:10.1161/01.RES.0000016960.61087.86.

[28] A. Baher, Z. Qu, A. Hayatdavoudi, S.T. Lamp, M.-J. Yang, F. Xie, S. Turner, A. Garfinkel, J.N. Weiss, Short-term cardiac memory and mother rotor fibrillation, Am. J. Physiol. - Heart Circ. Physiol. (2006). doi:10.1152/ajpheart.00944.2005.

[29] M.A. Bray, J.P. Wikswo, Use of topological charge to determine filament location and dynamics in a numerical model of scroll wave activity, IEEE Trans. Biomed. Eng. 49 (2002) 1086–1093. doi:10.1109/TBME.2002.803516.

[30] S. Rush, H. Larsen, A practical algorithm for solving dynamic membrane equations, IEEE Trans. Biomed. Eng. BME-25 (1978) 389–392. doi:10.1109/TBME.1978.326270.

[31] J. Sundnes, G.T. Lines, A. Tveito, An operator splitting method for solving the bidomain equations coupled to a volume conductor model for the torso, Math. Biosci. 194 (2005) 233–248. doi:10.1016/j.mbs.2005.01.001.

[32] A.J. Workman, K.A. Kane, A.C. Rankin, The contribution of ionic currents to changes in refractoriness of human atrial myocytes associated with chronic atrial fibrillation, Cardiovasc. Res. 52 (2001) 226–235. doi:10.1016/S0008-6363(01)00380-7.

[33] R.F. Bosch, X. Zeng, J.B. Grammer, K. Popovic, C. Mewis, V. Kühlkamp, Ionic mechanisms of electrical remodeling in human atrial fibrillation, Cardiovasc. Res. 44 (1999) 121–131. doi:10.1016/S0008-6363(99)00178-9.

[34] H. Zhang, C.J. Garratt, J. Zhu, A.V. Holden, Role of up-regulation of IK1 in action potential shortening associated with atrial fibrillation in humans, Cardiovasc. Res. 66 (2005) 493–502. doi:10.1016/j.cardiores.2005.01.020.

[35] M. Wilhelms, M.M. Maleckar, J.T. Koivumäki, O. Dössel, G. Seemann, Benchmarking electrophysiological models of human atrial myocytes, Front. Comput. Physiol. Med. 3 (2013) 487. doi:10.3389/fphys.2012.00487.

[36] D. Dobrev, U. Ravens, Remodeling of cardiomyocyte ion channels in human atrial fibrillation, Basic Res. Cardiol. 98 (2003) 137–148. doi:10.1007/s00395-003-0409-8.

[37] T. Osaka, Atsushiitoh, I. Kodama, Action potential remodeling in the human right atrium with chronic lone atrial fibrillation, Pacing Clin. Electrophysiol. 23 (2000) 960–965. doi:10.1111/j.1540-8159.2000.tb00881.x.
